# Comparative Analysis of Acute Kidney Injury Models and Related Fibrogenic Responses: Convergence on Methylation Patterns Regulated by Cold Shock Protein

**DOI:** 10.3390/cells13050367

**Published:** 2024-02-20

**Authors:** Sabine Brandt, Anja Bernhardt, Saskia Häberer, Katharina Wolters, Fabian Gehringer, Charlotte Reichardt, Anna Krause, Robert Geffers, Sascha Kahlfuß, Andreas Jeron, Dunja Bruder, Jonathan A. Lindquist, Berend Isermann, Peter R. Mertens

**Affiliations:** 1 Clinic of Nephrology and Hypertension, Diabetes and Endocrinology, Otto-von-Guericke University, 39120 Magdeburg, Germany; sabine.brandt@med.ovgu.de (S.B.); anja.bernhardt@med.ovgu.de (A.B.); saskiahaeberer@web.de (S.H.); fabian.gehringer@med.ovgu.de (F.G.); charlotte.reichardt@med.ovgu.de (C.R.); anna1.krause@st.ovgu.de (A.K.); jon.lindquist@med.ovgu.de (J.A.L.); 2 Medical Faculty, Health Campus Immunology, Infectiology and Inflammation (GCI-3), Otto-von-Guericke University, 39120 Magdeburg, Germany; sascha.kahlfuss@med.ovgu.de (S.K.); andreas.jeron@med.ovgu.de (A.J.); dunja.bruder@med.ovgu.de (D.B.); 3 Center for Health and Medical Prevention (CHaMP), Otto-von-Guericke University, 39120 Magdeburg, Germany; 4 Genome Analytics Research Group, Helmholtz Centre for Infection Research, 38124 Braunschweig, Germany; robert.geffers@helmholtz-hzi.de; 5 Institute of Molecular and Clinical Immunology, Otto-von-Guericke University, 39120 Magdeburg, Germany; 6 Institute of Medical Microbiology, Infection Control and Prevention, Otto-von-Guericke University, 39120 Magdeburg, Germany; 7 Research Group Immune Regulation, Helmholtz Centre for Infection Research, 38124 Braunschweig, Germany; 8 Institute of Laboratory Medicine, Clinical Chemistry and Molecular Diagnostics, University Hospital Leipzig, Leipzig University, 04103 Leipzig, Germany; berend.isermann@medizin.uni-leipzig.de

**Keywords:** acute kidney injury, matrisome, extracellular matrix, renal fibrosis, cold shock proteins, methylation, Klotho

## Abstract

Background: Fibrosis is characterized by excessive extracellular matrix formation in solid organs, disrupting tissue architecture and function. The Y-box binding protein-1 (YB-1) regulates fibrosis-related genes (e.g., *Col1a1*, *Mmp2*, and *Tgfβ1*) and contributes significantly to disease progression. This study aims to identify fibrogenic signatures and the underlying signaling pathways modulated by YB-1. Methods: Transcriptomic changes associated with matrix gene patterns in human chronic kidney diseases and murine acute injury models were analyzed with a focus on known YB-1 targets. *Ybx1*-knockout mouse strains (*Ybx1^ΔRosaERT+TX^* and *Ybx1^ΔLysM^*) were subjected to various kidney injury models. Fibrosis patterns were characterized by histopathological staining, transcriptome analysis, qRT-PCR, methylation analysis, zymography, and Western blotting. Results: Integrative transcriptomic analyses revealed that YB-1 is involved in several fibrogenic signatures related to the matrisome, the WNT, YAP/TAZ, and TGFß pathways, and regulates Klotho expression. Changes in the methylation status of the Klotho promoter by specific methyltransferases (DNMT) are linked to YB-1 expression, extending to other fibrogenic genes. Notably, kidney-resident cells play a significant role in YB-1-modulated fibrogenic signaling, whereas infiltrating myeloid immune cells have a minimal impact. Conclusions: YB-1 emerges as a master regulator of fibrogenesis, guiding DNMT1 to fibrosis-related genes. This highlights YB-1 as a potential target for epigenetic therapies interfering in this process.

## 1. Introduction

Chronic kidney disease (CKD) affects 8–15% of individuals worldwide, imposing a significant socioeconomic burden [1,2,3]. Following tissue injury, wound healing processes take place that coordinate regeneration or the accumulation of a substitute composed of fibrous collagens [4]. Fibrosis defines this progressive condition in solid organs, contributing to an estimated 45% of all deaths in the developed world [5]. In the kidney, fibrosis occurs as relentless loss of nephrons and the development of CKD. Excessive scarring and tissue damage within the kidneys impair their function, resulting in reduced filtration and overall organ dysfunction. Notably, the underlying pathomechanisms and molecular pathways of CKD appear to be shared irrespective of the primary inciting events [6]. Hitherto, there is no specific treatment option targeting fibrosis development per se, highlighting the urgent need to understand how fibrosis progresses. Given the commonalities of fibrosis irrespective of underlying injuries, our goal is to identify key decision points that may be amenable for therapeutic intervention.

The extracellular matrix (ECM) comprises more than 1.000 proteins. It undergoes continuous turnover to maintain tissue and cell homeostasis [7,8,9,10]. Its constituents are classified as core matrisome components comprising glycoproteins, collagens, and proteoglycans. Additionally, matrisome-associated or affiliated proteins, regulators, and secreted factors are also constituents [11]. In the context of the kidney, a subclassification of the matrisome into tubulointerstitial and glomerular components has been proposed [12,13,14]. This classification helps define specific cell damage events that trigger the secretion and activation of factors like transforming growth factor-β1 (TGFβ) and soluble factors like wingless/integrated (WNT), tumor necrosis factor (TNF), and sonic hedgehog (SHH) [15]. Recent advancements in single-cell transcriptome analysis provide unprecedented insights into dynamic and complex cellular events following kidney injury. This includes the formation of early cell niches referred to as fibrogenic within a specialized tissue microenvironment [6,16,17]. Components of these include tubular and endothelial cells, fibroblasts, pericytes, and immune cells. In-depth analyses unveiled the roles played by various soluble factors, such as WNT, TNF, and SHH, as well as extracellular vesicles and metabolites like glucose, lipids, and amino acids. These elements create a distinct environment within a specialized extracellular matrix scaffold [6,18,19,20]. 

ECM synthesis is regulated at both transcriptional and translational levels [21]. In the cytoplasm, multiprotein complexes store messenger RNA. These include transcripts for collagen and TGFβ, which may be rapidly translated for protein synthesis [22,23]. A prolonged half-life of collagen transcripts is critical for fibrosis development [24,25].

Gene transcription and translation are partly controlled by the cold-shock-domain-containing Y-box-binding protein (YB-1, gene: *Ybx1*), which belongs to the RNA-binding protein family [26]. YB-1 has multiple activities as a (i) classical transcription factor, (ii) a co-factor that alters the pattern of RNA splicing, (iii) an mRNA chaperone within ribonucleoprotein complexes which impact the mRNA pool and the immediate availability of untranslated mRNA, (iv) an extracellular mitogen/chemoattractant factor, and (v) a ligand for receptor signaling (Notch3, TNFR) [22,27,28]. YB-1 exhibits distinct pro- and anti-fibrotic functions depending on its subcellular localization and the type of expressing cell [29,30,31]. Anti-fibrotic activities of YB-1 have been observed for nuclear YB-1 in HEK293 cells in vitro as well as kidney tissue in vivo [29]. At the molecular level, YB-1 interferes with Smad2/3 signaling and collagen gene transcription [32]. Furthermore, the pharmacologically enforced nuclear translocation of cytoplasmic YB-1 significantly ameliorates organ fibrosis in the liver and kidney following injury [29,33]. 

Based on these observations, targeting YB-1 within kidney cells holds the promise of modulating organ fibrosis. To test this hypothesis, we established an inducible whole-body *Ybx1*-knockout strain that was subjected to acute kidney disease models (unilateral ureteral obstruction (UUO), nephrotoxic serum nephritis (NTS), and ischemia–reperfusion (I/R)) and compared the results to those of wild-type animals. Our comprehensive analyses of differentially regulated genes provide valuable insights, shedding light on the adherence and skewing of profibrogenic signaling signatures. Furthermore, by comparing our findings with those from another animal strain which specifically lacks the *Ybx1* gene in myeloid cells (*Ybx1^ΔLysM^*), we are able to dissect the contributions of tissue-resident cells versus infiltrating immune cells in the process of organ fibrosis. 

## 2. Materials and Methods

### 2.1. Microarray Data

GEO (http://www.ncbi.nlm.nih.gov/geo; accessed on 15 October 2023) [34] is a public functional genomics data repository of high-throughput gene expression data, chips, and microarrays. Two gene expression datasets (GSE30122 and GSE116626) were downloaded. The GSE30122 dataset contained nine glomerular tissue samples from patients with diabetic nephropathy (DN) and twenty-six glomerular tissue samples from control human kidneys [35]. The GSE116626 dataset contained twelve samples from patients with chronic lesions with IgA nephropathy and seven tissue samples from healthy living donors [36].

### 2.2. Animals

Both the *Ybx1^ΔLysM^* as well as the inducible whole-body *Ybx1*-knockout (*Ybx1^ΔRosaERT+TX^*) strains were established in our lab and described previously [37,38]. The animals were maintained according to the FELASA guidelines (*Federation of European Laboratory Animal Science Association*) on a 12 h/12 h light–dark cycle at 22 °C in the Animal Facility of the Otto-von-Guericke University Magdeburg under specific pathogen-free (SPF) conditions using individual ventilated cages (Tecniplast, Buguggiate, Italy) with free access to food and water. All procedures were conducted in accordance with the German National Guidelines for the Use of Experimental Animals (Animal Protection Act) and approved by the State of Saxony-Anhalt (AZ UniMD 42502-2-1213 and 42502-2-1293).

### 2.3. Genotyping

All mouse strains were confirmed by PCR genotyping. Briefly, the mouse tail was lysed with 200 µL of a direct PCR tail lysis buffer (Viagen, LA, USA) and 50 µg of proteinase K (Roche, Mannheim, Germany). The PCR primer pairs used for genotyping the mice are listed in Table 1. The procedure was as follows: Rosa26Cre PCR—35 cycles at 95 °C for 30 s, 67 °C for 30 s, and 72 °C for 120 s; Rosa26Cre-WT PCR—45 cycles at 95 °C for 30 s, 15 cycles at 61 °C, 58 °C, 55 °C for 30 s, and 45 cycles at 72 °C for 120 s; conditional YB-1-knockout PCR—35 cycles at 95 °C for 30 s, 60 °C for 30 s, and 72 °C for 60 s; and LysMCre PCR—36 cycles at 94 °C for 60 s, 56 °C for 30 s, and 72 °C for 120 s. Products were separated on 1% agarose DNA stain G gels (Serva, Heidelberg, Germany).

### 2.4. Experimental Kidney Disease

Unilateral ureter obstruction (UUO) was performed with gender-matched 12–16-week-old mice. The right ureters of *wild-type Ybx1^flox/flox^* mice (C57BL/6N) and *WT^RosaERT2Cre^* mice (C57BL/6J), as well as the *Ybx1*-knockout mice (*Ybx1^ΔRosaERT+TX^)* were ligated for 6 or 14 days. Contralateral kidneys were harvested for comparative analyses. Nephrotoxic serum nephritis was induced by a single intravenous injection of 200 µL of sheep anti-rat glomerular immunoglobulin preparation (Probetex) into *wild-type* and *Ybx1^ΔRosaERT+TX^* mice. Control mice were injected with PBS. Kidneys were analyzed at days 4 and 10 following injection. IRI was performed with gender-matched 9-to-14-week-old mice. Renal arteries and veins were occluded bilaterally with surgical clamps for 25 min. All animals received a subcutaneous analgesic at the end of the procedure. The mice were placed in a temperature-controlled environment during the recovery phase and returned to their cages with free access to food and water. In control animals, a mock operation was performed except for ischemia. Kidneys were harvested at days 1 and 28 following renal IRI. Both kidneys were collected for the preparation of cortical RNA, protein lysates, immunohistochemistry, and flow cytometry (FACS). Blood samples obtained via heart puncture were subjected to complete blood cell counting (ADVIA 120; Bayer Diagnostics, Munich, Germany), and plasma specimens were stored at −80 °C until further analysis. 

### 2.5. Analysis of Kidney Function

Urine was collected in metabolic cages (Tecniplast). For this, individual mice were placed in cages for 24 h with access to pelleted feed and fresh water ad libitum, and urine samples were collected. The urine volume was determined, and the samples were centrifuged (1500× *g*, 10 min). The protein content was determined using NobiStrip U10 test stripes (HI-309-38810601, Hitado, Möhnesee, Germany). Urea and creatinine values in serum samples were quantified using enzymatic assays (Roche Diagnostics, Mannheim, Germany, Cobas c501 module). 

GFR was measured in 3-month-old mice via the transcutaneous clearance of fluorescein isothiocyanate (FITC)–sinistrin using an NIC-Kidney Device (Mannheim Pharma & Diagnostics, Germany) [39].

### 2.6. Methylation Analysis

A methylation-specific PCR was performed as previously described [40,41]. Genomic DNA was isolated using the QIAamp DNA Mini Kit (QIAGEN, Hilden, Germany) and incubated with sodium bisulfite using the EZ DNA Methylation Kit (Zymo Research, Europe, Freiburg im Breisgau, Germany), following the manufacturer’s instructions. PCR amplification was performed with the EpiTect MSP Kit (Qiagen), using primer pairs designed to specifically detect either methylated or unmethylated CpG sites in the mouse Klotho promoter. The sequences of the primers used for methylated Klotho (mKL/Methylated), unmethylated Klotho (mKL/un-methylated, and input control are listed in Table 2 [42].

### 2.7. Isolation of Total RNA

Total RNA was extracted from kidney tissue samples using Trizol reagent (Life Technologies, Darmstadt, Germany) according to the manufacturer’s instructions. 

### 2.8. Quantitative Real-Time PCR

RNA was reverse-transcribed using RevertAid First Strand cDNA kits (Thermo Fisher Scientific, Waltham, MA, USA). A quantitative PCR was performed using SYBR Green PCR Master Mix (Thermo Fisher Scientific, Waltham, MA, USA) on a 7500 Fast Real Time PCR TaqMan sequence detector (Applied Biosystems, Darmstadt, Germany). GAPDH was used as an internal control, and relative mRNA levels were normalized to the control sample using the ΔΔCt method. The sequences of the primers used for the real-time PCR are listed in Table 3.

### 2.9. Gene Array

RNA extraction from kidney tissue lysates and gene array analyses have been described [37,43]. RNA was quantified using a NanoDrop-1000 spectrophotometer, and quality was monitored with an Agilent 2100 Bioanalyzer (Agilent Technologies, Santa Clara, CA, USA). Labeling protocol: Cyanine 3′-labeled cRNA was prepared from 0.5 μg of RNA using a One-Color Low RNA Input Linear Amplification PLUS kit (Agilent Technologies, Santa Clara, CA, USA) according to the manufacturer’s instructions, followed by RNAeasy column purification (Qiagen, Hilden, Germany). Dye incorporation and cRNA yield were checked using a NanoDrop ND-1000 Spectrophotometer. Hybridization protocol: Cyanine 3′-labeled cRNA (1.5 μg; specific activity: >10.0 pmol cyanine 3/μg cRNA) was fragmented at 60 °C for 30 min in a reaction volume of 250 mL containing 1X Agilent fragmentation buffer and 2X Agilent blocking agent, following the manufacturer’s instructions. Upon completion of the fragmentation reaction, 250 mL of 2X Agilent hybridization buffer was added to the fragmentation mixture and hybridized using Agilent 4 × 44 k Mouse V2 Design ID: 026,655 for 17 h at 65 °C in a rotating Agilent hybridization oven. After hybridization, the microarrays were washed for 1 min at room temperature with GE Wash buffer 1 (Agilent Technologies, Santa Clara, CA, USA) and for 1 min with 37 °C GE Wash buffer 2 (Agilent Technologies, Santa Clara, CA, USA); they were then dried immediately via brief centrifugation. Data processing: the scanned images were analyzed using Feature Extraction Software 10.5 (Agilent Technologies, Santa Clara, CA, USA), using default parameters to obtain background-subtracted and spatially detrended processed signal intensities. 

### 2.10. Transcriptome Analysis Using RNA-Seq

The quality and integrity of the total RNA was controlled on an Agilent Technologies 2100 Bioanalyzer (Agilent Technologies; Waldbronn, Germany). The RNA sequencing library was generated from 500 ng of total RNA using a Dynabeads^®^ mRNA DIRECT™ Micro Purification Kit (Thermo Fisher) for mRNA purification, followed by an NEBNext^®^ Ultra™ II Directional RNA Library Prep Kit (New England BioLabs) according to the manufacturer’s protocols. The libraries were sequenced on an Illumina NovaSeq 6000 using a NovaSeq 6000 S1 Reagent Kit (100 cycles; paired-end run), with an average of 3 × 10^7^ reads per RNA sample. Each FASTQ file received a quality report generated by the FASTQC tool. Before its alignment to the reference genome, each sequence in the raw FASTQ files was trimmed on base call quality and sequencing adapter contamination using the Trim Galore wrapper tool. Reads shorter than 20 bp were removed from the FASTQ file. Trimmed reads were aligned to the reference genome using the open-source short-read aligner STAR (https://code.google.com/p/rna-star/; accessed on 15 October 2023) with settings according to the log file. Feature counts were determined using the R package “Rsubread”. Only genes showing counts greater than 5 at least two times across all samples were considered for further analysis (data cleansing). Gene annotation was carried out using the R package “bioMaRt. Before starting the statistical analysis, expression data were log2 transformed and normalized according to TMM normalization using the edgeR package. The R package “edgeR” calculates differential gene expression. 

### 2.11. Tissue Lysate Preparation and Western Blot Analysis

Kidney tissue was mechanical homogenized in a RIPA buffer (50 mM Tris–HCl, 150 mM Nonidet *p*-40, 1 mM sodium deoxycholate, 1 mM EDTA, and 1 mM sodium orthovanadate) containing a complete protease inhibitor cocktail (Roche, Mannheim, Germany) at 4 °C for 15 min. Protein contents were determined using the Bio-Rad protein assay (Bio-Rad, Munich, Germany). Denatured protein samples were separated using 10% SDS-PAGE and blotting onto nitrocellulose membranes. The membranes were blocked with 5% dry milk in TBS/Tween and incubated with primary antibodies diluted in TBS/T overnight at 4 °C. The following antibodies were used: anti-YB-1_C-terminal_ (Y0396, Sigma, Taufkirchen, Germany), α-GAPDH (14C10, Cell Signaling, Frankfurt am Main, Germany), and α-Klotho (AF1819, R&D). This was followed by incubation with the secondary HRP-conjugated goat anti-rabbit IgG (Southern Biotech, Birmingham, AL, USA) or donkey anti-goat IgG (Jackson Immuno Research, Cambridge House, UK) for 30 min. Following additional washing steps, a SuperSignal chemiluminescence substrate, Pierce ECL (Thermo Scientific, Darmstadt, Germany), was added, and emitted light was measured using an Intas imaging system.

### 2.12. Gelatin Zymography

Equal quantities of protein from kidney cortex homogenates were loaded and electrophoresed using 10% novex zymogram gelatin gels with a Tris–Glycine SDS Running Buffer (Thermo Fisher, Darmstadt, Germany) under a constant voltage of 125 V. Enzymes were renatured and developed, followed by staining with Coomassie Blue R-250 (Bio-Rad, Munich, Germany). The gelatinolytic activity of MMP2 was visualized following destaining using clear white bands on a blue background. Band intensities were quantified using Lab-Image Software. 

### 2.13. Immunohistochemistry

Methacarn-fixed, paraffin-embedded tissue sections were used for immunohistochemistry (using the avidin–biotin complex (ABC) method). For dewaxing, the sections were placed for 10 min in xylene, followed by a descending alcohol series and immersion with distilled water for 5 min. For Klotho and fibrinogen staining, specimens were heated in a 10 mM sodium citrate buffer (pH 6.0) in a microwave for 5 min. Endogenous peroxidase was inactivated by incubation with 3% hydrogen peroxide. After washing, the sections were incubated with a blocking solution (10% FCS, 1% BSA in 1× Tween–PBS) for 30 min at room temperature, and a primary antibody (goat anti-mouse KLOTHO, R&D Systems, AF1819; goat anti-mouse Fibrinogen, Santa Cruz, 18032) was added overnight at 4 °C diluted in 1% FCS plus 1× Tween–PBS. After incubation with a peroxidase-labeled secondary antibody (Jackson Immuno Research, 705-035-147, 1:500) for 1 h at room temperature, the sections were developed using a diaminobenzidine substrate and counterstained with a hematoxylin solution. Subsequent dehydration was achieved using ascending alcohol series (70% ethanol, 80% ethanol, 96% ethanol, and 100% ethanol). Finally, washing was performed twice for 5 min in Histo-Clear (Carl Roth, Karlsruhe, Germany). Embedding was performed with a ROTI-Histokitt II (Carl Roth, Karlsruhe, Germany), according to the manufacturer’s instructions.

### 2.14. Periodic Acid–Schiff (PAS) Staining

Sections were deparaffinized using xylene, followed by a descending alcohol series and immersion with distilled water for 5 min. For staining, the sections were incubated with a periodic acid solution and Schiff reagent (both from Sigma-Aldrich, Taufkirchen, Germany), followed by an extensive washing step. The counterstain was carried out with a hematoxylin solution. Subsequently, dehydration was achieved by means of an ascending alcohol series (70% ethanol, 80% ethanol, 96% ethanol, and 100% ethanol). Finally, washing was performed twice for 5 min in Histo-Clear (Carl Roth, Karlsruhe, Germany). Embedding was performed with a ROTI-Histokitt II (Carl Roth, Karlsruhe, Germany), according to the manufacturer’s instructions.

### 2.15. Sirius Red Staining

Deparaffinized sections were incubated with a 0.1% Sirius red solution in saturated picric acid (Chroma, Ooching, Germany) and were destained with 0.01N hydrochloric acid. Subsequent dehydration was achieved by means of an ascending alcohol series (70% ethanol, 80% ethanol, 96% ethanol, and 100% ethanol). Finally, washing was performed twice for 5 min in Histo-Clear (Carl Roth, Karlsruhe, Germany). Embedding was performed with a ROTI-Histokitt II (Carl Roth, Karlsruhe, Germany).

### 2.16. Bioinformatic Analysis

Each list of differentially expressed genes derived from the respective comparisons was subjected to a functional and biochemical pathway analysis using G:Profiler, a web server for functional enrichment analyses and conversions of gene lists. Enrichment blots were generated in Python using the Matplotlib library and Spyder programming environment. Volcano plots were generated using GraphPad Prism 8.0 software (GraphPad Software, La Jolla, CA, USA). 

The expression of matrisome genes previously described was analyzed with the MatrisomeDB gene collection [11].

### 2.17. Statistical Analysis

All results were confirmed through at least two independent experiments performed in triplicate unless otherwise stated. Results were calculated and are presented as mean ± standard deviation (SD) values. Statistical analyses were performed using GraphPad Prism 8.0 software (GraphPad Software, La Jolla, CA, USA) and a two-way ANOVA followed by Sidak’s multiple-comparisons test. Student’s *t*-test was applied for two-group comparisons, with *p* < 0.05 (*), *p* < 0.01 (**), and *p* < 0.001 (***) considered statistically significant.

## 3. Results

### 3.1. Transcriptional Programs for Kidney Fibrosis in Chronic Kidney Disease

**Figure 1:**
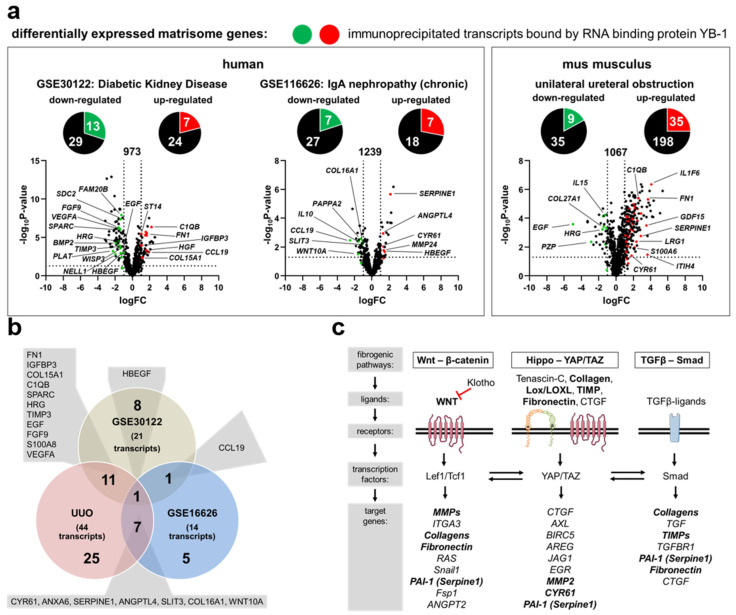
Differentially expressed genes encoding for matrisome proteins in diseased kidneys are regulated by *Ybx1*. (**a**) Volcano plots depict differentially regulated genes belonging to the matrisome between healthy and diseased kidneys as well as disease entities shown as log_2_(fold change) values on the *x*-axis and –log_10_(*p*-value) values on the *y*-axis. The red (up-regulated) and green (down-regulated) portions of the circles represent YB-1-associated transcripts (according to Finkbeiner et al., 2009 [44]). Detailed information about gene names, accession numbers, and functions is listed in Supplementary Table S2. (**b**) A comparison of the differentially regulated genes in diseased human kidneys and the murine UUO model. There is an overlap of 20 genes within the datasets. (**c**) A simplified schematic overview of three major fibrogenic pathways. Transcripts identified by YB-1 pulldown are highlighted in bold.

In order to identify changes in matrix-dependent gene expression, two publicly accessible CKD patient data sets in the Gene Expression Omnibus (GEO) database were analyzed [34]. The data sets are described in Supplementary Table S1. Differentially expressed genes (DEGs) from both data sets are shown in Supplementary Table S2. Integrative analyses were performed to identify overlap between the DEGs and the published matrisome database [11]. A total of 73 matrisome-dependent DEGs were identified in patients with diabetic kidney disease. Out of these, 42 genes were down-regulated and 31 were up-regulated (Figure 1a, Supplementary Table S2). Similar relationships were seen in kidney samples from patients diagnosed with IgA nephropathy. Out of 59 DEGs, 34 were down-regulated and 25 were up-regulated (Figure 1a). A further comparison of YB-1-associated genes [44] and matrisome gene expression demonstrated an overlap (Figure 1a). In both disease models, 25% of the genes assigned to the matrisome were associated with YB-1 (Table 4). Similar analyses were performed using kidneys from mice that underwent acute injury following unilateral ureteral obstruction (UUO), a model of tubulointerstitial damage with secondary glomerulosclerotic lesions (Figure 1a). An induction of YB-1 expression following UUO on both the single cells and protein levels has been reported before [29,37,45]. A comparison of the matrisome-specific and YB-1-related DEGs revealed an overlap of 20 genes within the human and mouse datasets (Figure 1b). Based on published signaling pathways of fibrosis, ~50% of the involved proteins/molecules are regulated by YB-1 (indicated by bold letters in Figure 1c) [46]. 

### 3.2. YB-1 Confers a Profibrogenic Response

**Figure 2:**
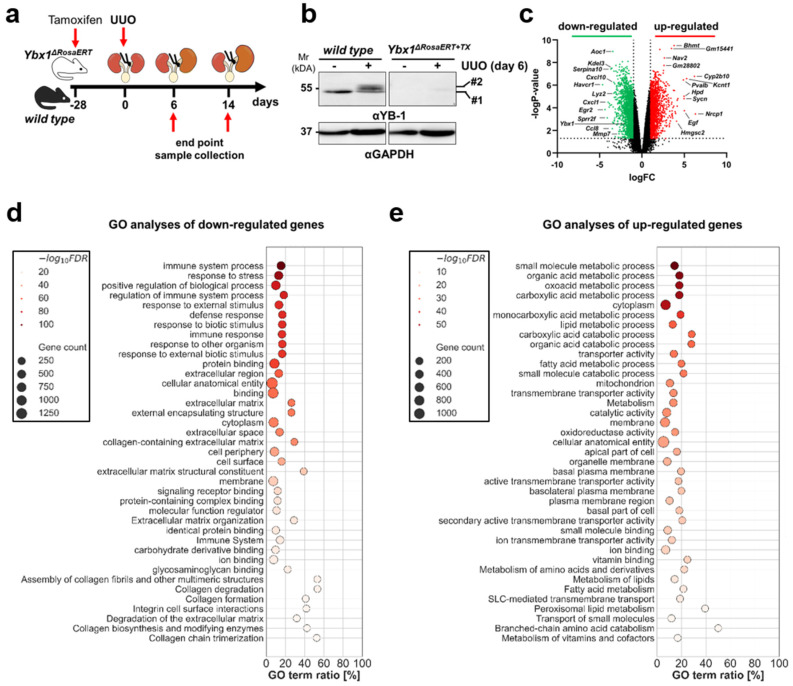
Following UUO, *Ybx1*-deficient kidneys express fewer matrix genes compared to wild-type animals. (**a**) Experimental design with unilateral ureteral obstruction in wild-type (n = 3–6) and *Ybx1^ΔRosaERT+TX^* (n = 3–6) mice following tamoxifen application. Three-month-old mice were gavaged with tamoxifen 28 days before UUO to induce *Ybx1* knockout. (**b**) Representative immunoblots of YB-1 protein expression in contralateral or UUO day 6 kidneys from wild-type and *Ybx1^ΔRosaERT+TX^* animals. Here, #1 represents YB-1 with the expected size at 50 kDa; and #2 indicates that following UUO, there is a slight increase in the relative molecular weight of YB-1. (**c**) Volcano plot representing the differentially expressed genes in wild-type versus *Ybx1^ΔRosaERT+TX^* animals following UUO (day 6), shown as log2(fold change) values on the x-axis and –log10(*p*-value) values on the y-axis. Transcripts depicted in green were significantly down-regulated, while transcripts depicted in red were significantly up-regulated in *Ybx1^ΔRosaERT+TX^* compared to wild-type mice. Significantly regulated transcripts were defined those with logFC ≤ ±1 and −log*p*-value > 1.3 (dotted lines). (**d**,**e**) A gene enrichment analysis using G:Profiler was performed, and scatter plots of the top down-regulated (**d**) or up-regulated (**e**) pathways in *Ybx1^ΔRosaERT+TX^* mice were compared to wild-type mice based on GO molecular function, biological process, cellular composition, and reactome databases. The sizes of the dots reflect the gene counts for each process, and the dot color reflects the adjusted *p*-values; the most significant result is purple.

We took advantage of a tamoxifen-inducible *Ybx1*-knockout model [38] to define its role in ECM composition and abundance (Supplementary Figure S1a). No differences between wild-type and *Ybx1^ΔRosaERT+TX^* knockout littermates were seen in kidney morphology and function (blood urea nitrogen (BUN); serum creatinine (Supplementary Figure S1b,c)). Long-term observation beyond 2 years of age shows no phenotypic abnormalities in the *Ybx1^ΔRosaERT+TX^* knockout mice except for thinner skin and abnormalities of the fur (e.g., hair loss) [38]. Kwon et al. described a decline in epidermal thickness and decreases in developed hair follicles and Ki67-positive cells in vivo at E.16 in *Ybx1*-knockout animals [47]. A transcriptome analysis confirmed no changes in matrisome composition between the kidneys of healthy wild-type and *Ybx1*-knockout animals (Supplementary Figure S1d,e). The kidneys of wild-type and tamoxifen-inducible *Ybx1*-knockout mice with UUO (time interval: 6 days post ligation) and sham-operated mice were harvested to generate Agilent MicroArray libraries (Figure 2a,b). A fibrotic signature was seen in wild-type and knockout animals, albeit to different degrees (Supplementary Figure S2a,b). In the diseased kidneys of wild-type and *Ybx1^ΔRosaERT+TX^* knockout animals 3700 DEGs were identified (Figure 2c). A gene set enrichment analysis revealed that the most significantly down-regulated genes in the *Ybx1^ΔRosaERT+TX^* knockout versus wild-type animals relate to organ fibrosis and inflammation (Figure 2d). The up-regulated genes are part of metabolic processes and transmembrane transport activities (Figure 2e). 

**Figure 3:**
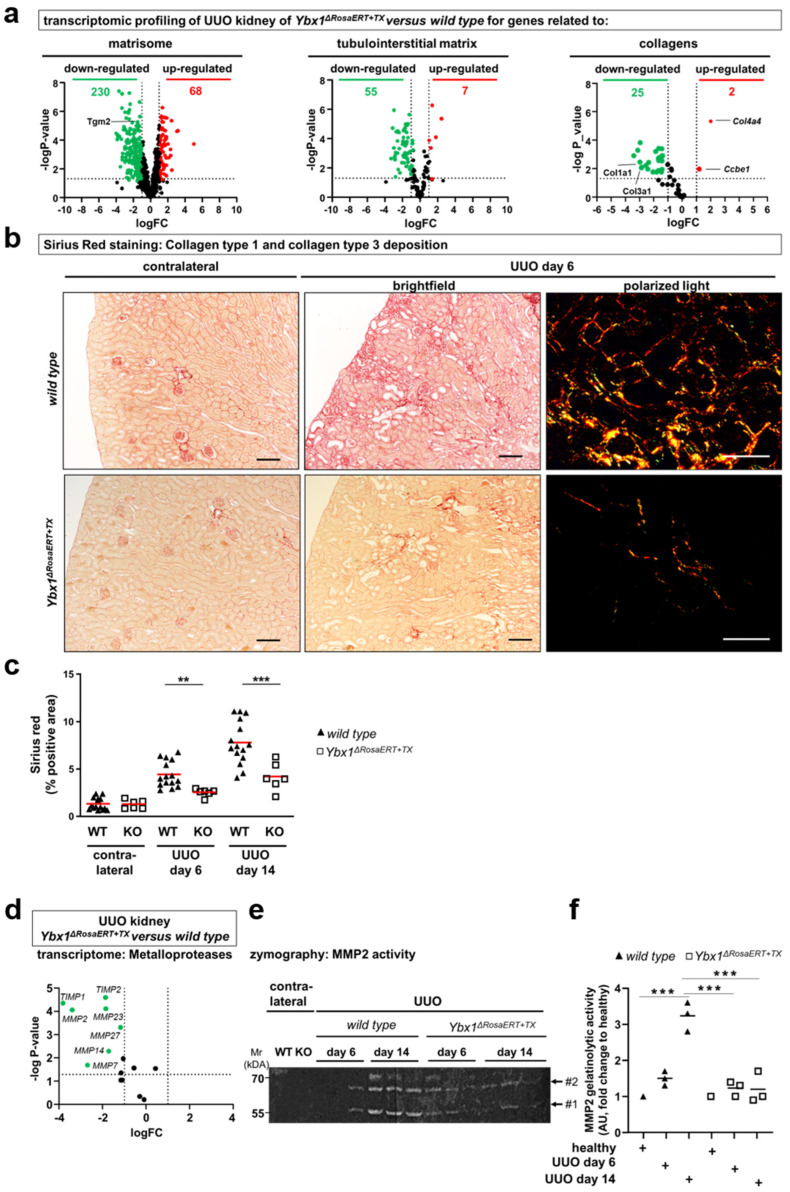
Following UUO, matrisome-defining genes are less expressed in *Ybx1*-deficient kidneys compared to wild-type animals. (**a**) Total kidney RNA from healthy and diseased kidneys on day 6 following UUO was isolated, and a transcriptome analysis was performed. Volcano plots visualizing differential mRNA abundance shown as log2(fold change) values of healthy over diseased kidneys on the x-axis and –log10(*p*-value) values on the y-axis. Genes defining the matrisome, tubulointerstitial matrix, and collagens were analyzed in the kidneys of *Ybx1^ΔRosaERT+TX^* compared to wild-type animals. The majority of the transcripts were less abundant in diseased *Ybx1^ΔRosaERT+TX^* kidneys compared to wild-type kidneys. The differences were particularly pronounced within the family of collagens. Significantly regulated transcripts were those with logFC ≤ ±1 and −log*p*-value > 1.3 (dotted lines). (**b**) Tissue sections of healthy (contralateral) and UUO kidneys were stained with Picro Sirius red. The deep red fibers represent collagen 1- and 3-positive areas. Further, polarized light enabled us to determine the ratio of collagen type I (red to orange) and type III (green). Representative images are shown. Scale bar, 50 µm. (**c**) Quantification of collagen deposition. (**d**) Gene expression analysis of differentially regulated matrix metalloproteinase (MMPs) and tissue inhibitors of metalloproteinases (TIMPs) in the kidneys of wild-type and *Ybx1^ΔRosaERT+TX^* animals following ureteral obstruction. MMP2 is the most differentially regulated matrix metalloproteinase. Significantly regulated transcripts were those with logFC ≤ ±1 and -log*p*-value > 1.3 (dotted lines). (**e**) Gelatin zymography was used to analyze MMP2 enzymatic activity in kidney tissue lysates of wild-type and Ybx1ΔRosaERT+TX animals. (#1, active MMP2; #2, latent MMP2). (**f**) Quantification of MMP2 enzymatic activity in wild-type compared to *Ybx1^ΔRosaERT+TX^* kidney tissue lysates. (** *p* < 0.01 and *** *p* < 0.001).

In a similar comparison, genes defining the matrisome composition were analyzed (Figure 3) [11,14]. In addition to the well-known targets of YB-1 (e.g., *Col1a1*, *Mmp2*, and *Tgfb1*) more than 100 genes (e.g., *Serpine1*, *Egr1*, *Timp1*, *Sparc1*, and *Postn*) were identified that are less abundant in *Ybx1^ΔRosaERT+TX^* knockout compared to wild-type animals. These are associated with tubulointerstitial fibrosis and extracellular matrix accumulation. Thus, a distinct wound healing process takes place within *Ybx1^ΔRosaERT+TX^* knockout animals.

Differences were particularly pronounced within the collagen protein family. *Col1a1* transcripts in the kidneys of the *Ybx1^ΔRosaERT+TX^* animals following UUO were ~2-fold less abundant than in wild-type animals (Figure 3a, Supplementary Table S3). The expression of transglutaminase-2, a known collagen crosslinker, was induced in the kidneys of wild-type mice and reduced in the *Ybx1^ΔRosaERT+TX^* mice. In accordance, collagen fiber visualization by Sirius red staining confirmed the blunted fibrogenic response in *Ybx1^ΔRosaERT+TX^* animals (Figure 3b,c). These findings prompted us to further focus on MMP2, a key player implicated in matrix degradation and kidney fibrosis, whose activity is intricately regulated by YB-1 expression [48]. 

### 3.3. MMP2 Activity following UUO Is Dependent on YB-1 Expression

In healthy tissue, the extracellular matrix undergoes constant remodeling, with an equilibrium between synthesis and degradation [49]. MMP2 drew our attention as it is implicated in the initiation and progression of kidney fibrosis and its gene transcription is regulated by YB-1 [48,50]. The UUO animals exhibited enhanced MMP2 gene expression and enzymatic activity in wild-type strains (Figure 3e,f). In contrast, *Ybx1^ΔRosaERT+TX^* knockout animals displayed fewer MMP2 transcripts (Figure 3d) and reduced enzymatic activity (Figure 3e,f) compared to wild-type animals, especially on day 14 of ureteral obstruction. The tissue inhibitors of metalloproteinases (TIMPs), such as *TIMP1* and *TIMP2*, were up-regulated in the kidney tissue of wild-type animals following UUO; however, this occurred to a much lesser degree in *Ybx1^ΔRosaERT+TX^* animals. In conclusion, the data suggest that in the absence of *Ybx1* gene expression, the overall gelatinolytic activity is decreased. This is counterintuitive given the decreased fibrogenesis in knockout animals. One explanation may be based on the lower collagen synthesis rate in knockout animals. Furthermore, some studies link MMP2 activities with a profibrogenic phenotype of kidney cells such as mesangial cells [51]. 

### 3.4. Key Fibrogenic Mechanisms Are Deregulated in YB-1-Deficient Kidneys 

**Figure 4:**
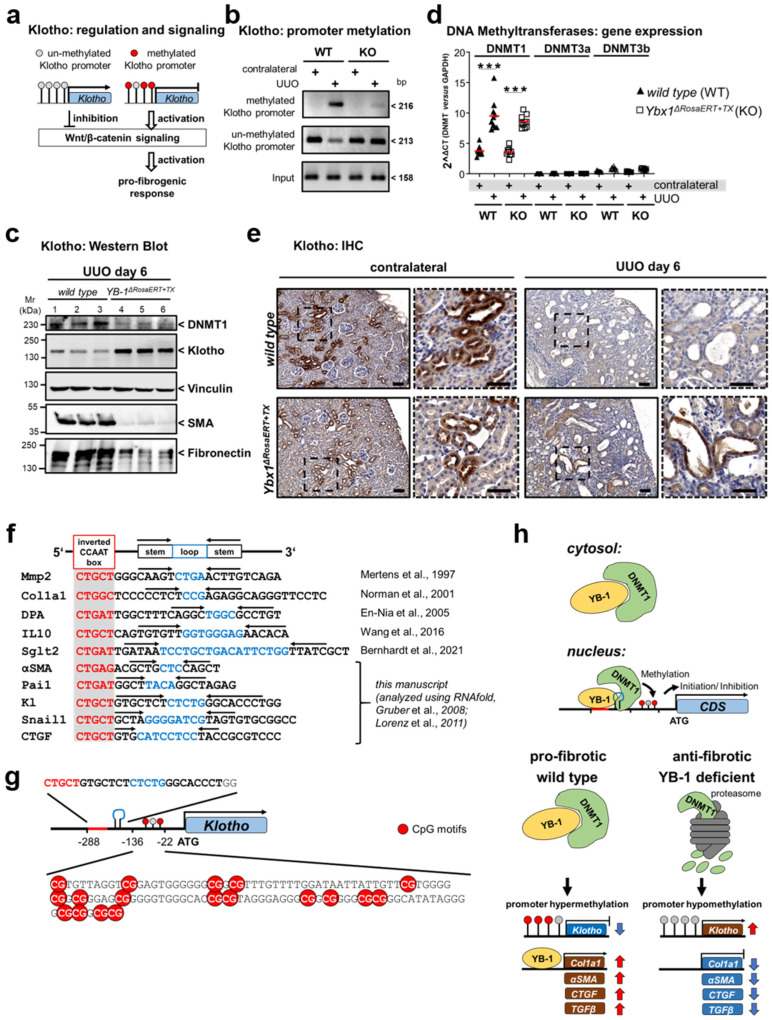
Klotho promoter hypermethylation and suppressed protein expression following UUO are abolished in *Ybx1*-deficient kidneys following UUO. (**a**) Diagram illustrating that Klotho promoter hypermethylation subsequently repressed expression, the WNT/β-catenin signaling pathway, and fibrosis. (**b**) Representative methylation-specific PCR of the Klotho promoter from contralateral and UUO mouse kidneys. (**c**) Expression of Klotho, αSMA, fibronectin, and DNMT1 in wild-type and *Ybx1^ΔRosaERT+TX^* mouse kidneys (3 samples from each group are shown). (**d**) Transcript levels of DNMT1, 3a, and 3b assayed by RT-PCR. (**e**) Representative immunohistochemistry of Klotho in healthy and diseased kidney tissues of wild-type and *Ybx1^ΔRosaERT+TX^* animals. (**f**) A comparison of Pai1, Kl (Klotho), Snail1, CTGF, and αSMA with known YB-1-binding motifs (red box) of matrix metalloproteinase2 (Mmp2), Col1a1, DNA polymerase-a (DPA), IL10, and Sglt2 genes reveals homologies and stem-loop motifs (indicated by arrows). (**g**) Schematic view of the Klotho promoter, including the YB-1 binding motif, CpG islands, and transcriptional start (ATG). (**h**) Under healthy conditions, the YB-1-DNMT1 complex is located in the cytosol. Following pro-fibrogenic stress, the complex translocates to the nucleus, where it binds to promoter regions such as Klotho, leading to promoter hypermethylation and the reduced expression of Klotho. The expression of the pro-fibrogenic genes Col1a1, αSMA, CTGF, and TGFβ is induced. A different scenario takes place in the kidney tissue of *Ybx1^ΔRosaERT+TX^* animals. No methylation of the aforementioned genes takes place due to the lack of DNMT1 [29,38,48,54,55,56,57], (*** *p* < 0.001).

In the course of UUO, a reduction in Klotho expression and signaling is observed in wild-type animals [45]. Recent studies suggest an association of incipient renal fibrosis with reduced Klotho expression via the WNT/β-catenin signaling pathway (summarized in Figure S4a). Mechanistically, Klotho expression is regulated by epigenetic modifications [46,52]. The Klotho promoter contains abundant CpG islands where methylation takes place. We performed methylation-specific polymerase chain reactions (PCRs) to identify CpG modifications (Figure 4b). In the non-diseased tissue of *wild-type* mice, the promoter is less methylated; however, it undergoes abundant methylation following UUO. The diseased tissue of *Ybx1^ΔRosaERT+TX^* animals exhibits a low degree of methylation, suggesting that YB-1 regulates the methylation of Klotho (Figure 4b). In line with epigenetic regulation, reduced Klotho transcript numbers and protein expression were seen in wild-type kidney tissue (Figure 4c,e, Supplementary Figure S3). At the same time, SMA and fibronectin protein levels were lowered. Following UUO in *Ybx1^ΔRosaERT+TX^* animals, the Klotho transcript numbers remained at high levels, as did protein expression (Figure 4c,e, Supplementary Figure S3). This suggests a direct link of YB-1 with Klotho transcriptional regulation. One possible mechanism could be via DNA methyltransferases. Since DNA methylation is executed by DNA methyltransferases (DNMTs), we quantified their levels (DNMT1, DNMT3a, and DNMT3b). Following UUO, there is increased expression of DNMT1 in both genotypes on the transcript level (Figure 4d). On the protein level, there is increased expression of DNMT1 in wild-type tissue following UUO; however, this was not observed in *Ybx1^ΔRosaERT+TX^* mice (Figure 4c), suggesting YB-1-dependent recruitment of DNMT1 to DNA. Expanding upon this hypothesis, we conducted a promoter sequence analysis of α*SMA*, *Pai1*, *Kl*, *Snail1*, and *CTGF,* genes known to be influenced by YB-1 (Figure 1c) [53]. All promoters contain a Y-box motif (an inverted CCAAT box) followed by a predicted stem-loop structure. Both are required for YB-1 binding to DNA (see Figure 4f). A further analysis uncovered multiple CpG motifs in close proximity (Figure 4g). We propose that YB-1 serves as a versatile regulator of fibrogenic gene expression by virtue of its ability to bind specific structural motifs within DNA, consequently facilitating the recruitment of methyltransferases. Under healthy conditions, the YB-1-DNMT1 complex is located in the cytosol. Following cell stress, the complex translocates to the nucleus, where it binds to promoter regions such as Klotho, leading to promoter hypermethylation and the reduced expression of Klotho. A different scenario takes place in the kidney tissue of *Ybx1^ΔRosaERT+TX^* animals. No methylation of the aforementioned genes takes place, possibly due to the lower abundance of DNMT1 (Figure 4h). Thus, we identified a further mechanism through which YB-1 regulates fibrogenic responses by regulating the epigenetic modification of the Klotho gene.

**Figure 5:**
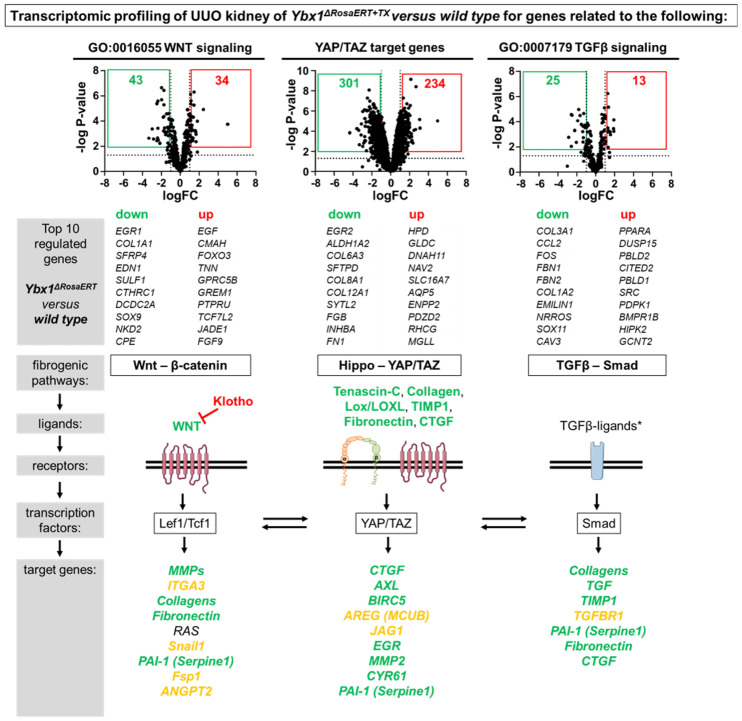
Key profibrotic signaling cascades are regulated by YB-1. Differential gene expression analysis of WNT signaling components, YAP/TAZ target genes, and TGFβ signaling components in the kidneys of wild-type and *Ybx1^ΔRosaERT+TX^* animals that underwent ureteral obstruction (tissue analyzed on UUO day 6). The top 10 down- and up-regulated genes are listed and the expression levels of the profibrotic signaling components are visualized using green for down-regulated genes in *Ybx1^ΔRosaERT+TX^* compared to wild-type kidney tissue, red for up-regulated genes, and orange for a lack of significant regulation between wild-type and *Ybx1^ΔRosaERT+TX^* Animals. * The TGFβ ligands have not been analyzed. The expression values of the transcription factors Lef1/Tcf1, YAP/TAZ, and Smad have not been evaluated.

As outlined in Figure 1c, the transcripts of key molecules related to fibrogenic pathways (WNT, TGFβ, and integrin) are regulated by the YB-1 protein. A differential gene expression analysis of WNT signaling (GO:0016055), YAP/TAZ target genes [58], and TGFß signaling (GO:0007179) components revealed that YB-1 may promote these pro-fibrogenic signaling cascades independently (Figure 5). The activation of these pathways at the same time raises the question as to whether this is a disease-specific phenomenon or a generalized effect mediated by cellular YB-1 activation. To answer this question, two different experimental models were analyzed in detail: the ischemia–reperfusion (I/R) injury with primarily tubular injury and nephrotoxic serum nephritis (NTS) with glomerular injury.

### 3.5. Similar Fibrogenic Pathways Are Regulated by YB-1 in a Glomerular Injury Model as Well as Following Ischemia–Reperfusion Injury

**Figure 6:**
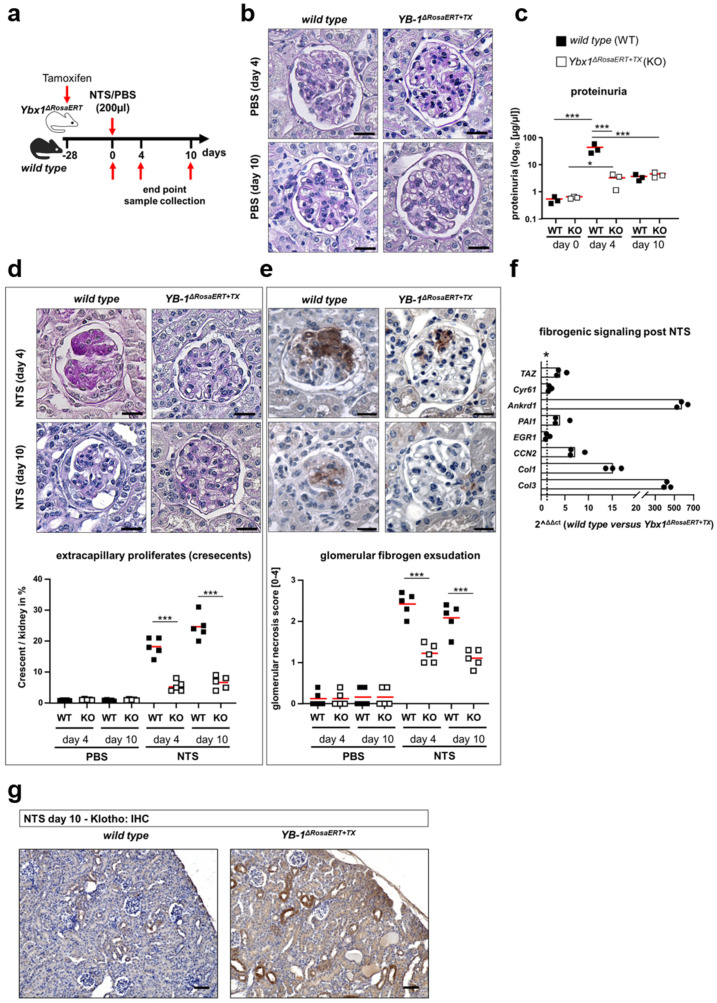
The fibrogenic pathways in the glomerular injury model are primarily regulated by YB-1. (**a**) Experimental design to induce nephrotoxic serum nephritis in wild-type (n = 3–5) and *Ybx1^ΔRosaERT+TX^* (n = 3–5) mice following tamoxifen application. Three-month-old mice were gavaged with tamoxifen 28 days before NTS application to induce Ybx1 knockout. Nephrotoxic serum nephritis (NTS) was induced by the injection of sheep anti-rat glomerular immunoglobulins. Kidneys were analyzed on days 4 and 10 following disease induction. (**b**) No structural differences are seen between the healthy glomeruli of wild-type and *Ybx1^ΔRosaERT+TX^* knockout mice, visualized by PAS staining. Scale bar, 50 µm. (**c**) Proteinuria were analyzed before and on days 4 and 10 following NTS injection. (**d**) Wild-type animals developed enhanced glomerular injury with the formation of extracapillary proliferates (crescents) as well as (**e**) enhanced fibrinogen deposition and exudation compared to *Ybx1^ΔRosaERT+TX^* knockout mice following NTS injection. (**f**) Within the kidney tissue of wild-type mice, the transcripts of TAZ and the target genes thereof were increased following NTS injection compared to control kidneys. Amongst these, PAI1, Ankrd1, and CCN2 were less abundant in the kidneys of *Ybx1^ΔRosaERT+TX^* knockout mice compared to wild-type animals following NTS. The Cyr61 and EGR1 transcript numbers were similar in both animal models. (* values > 1 represent significant changes) (**g**) Representative renal immunohistochemistry staining of Klotho in healthy and diseased kidney tissues of wild-type and *Ybx1^ΔRosaERT+TX^* animals. (*** *p* < 0.001).

The (NTS) model incites an acute inflammatory response, immune cell infiltration, high-grade proteinuria, and glomerular scarring with subsequent fibrosis of periglomerular tissue [59]. These responses were not detected in *Ybx1^ΔRosaERT+TX^* knockout mice following NTS injection (Figure 6). Similar to the UUO model, we observed a reduction in Klotho expression in the kidney tissue of wild-type animals. In *Ybx1^ΔRosaERT+TX^* knockout mice, there is no reduction in Klotho (Figure 6). The deposition of sheep IgG on glomeruli is similar in both genotypes (Supplementary Figure S4). A pathway analysis of injured podocytes following NTS application revealed an up-regulation of signaling pathways, including Hippo, TGF-β, NFκB, and FoxO [59]. These changes are comparable to the UUO-dependent fibrogenic response. Within the kidney tissue of wild-type mice, the transcripts of the Hippo-YAP/TAZ-TGFβ target genes *Ankrd1*, *Cyr61*, *Egr1*, *PAI1*, and *CCN2* are more abundant following NTS injection when compared to healthy kidneys (Figure 6f). In the kidneys of *Ybx1^ΔRosaERT+TX^* knockout mice, amongst these, *PAI1, Ankrd1*, and *CCN2* were less abundant compared to wild-type animals following NTS, whereas *Cyr61* transcripts are regulated similarly in both animal models. In conclusion the Hippo pathway, especially *YAP*, *PAI1*, *Ankrd1*, and *CCN2*, is activated in a YB-1-dependent manner, confirming the pro-fibrogenic role of YB-1 outlined in Figure 1c.

**Figure 7:**
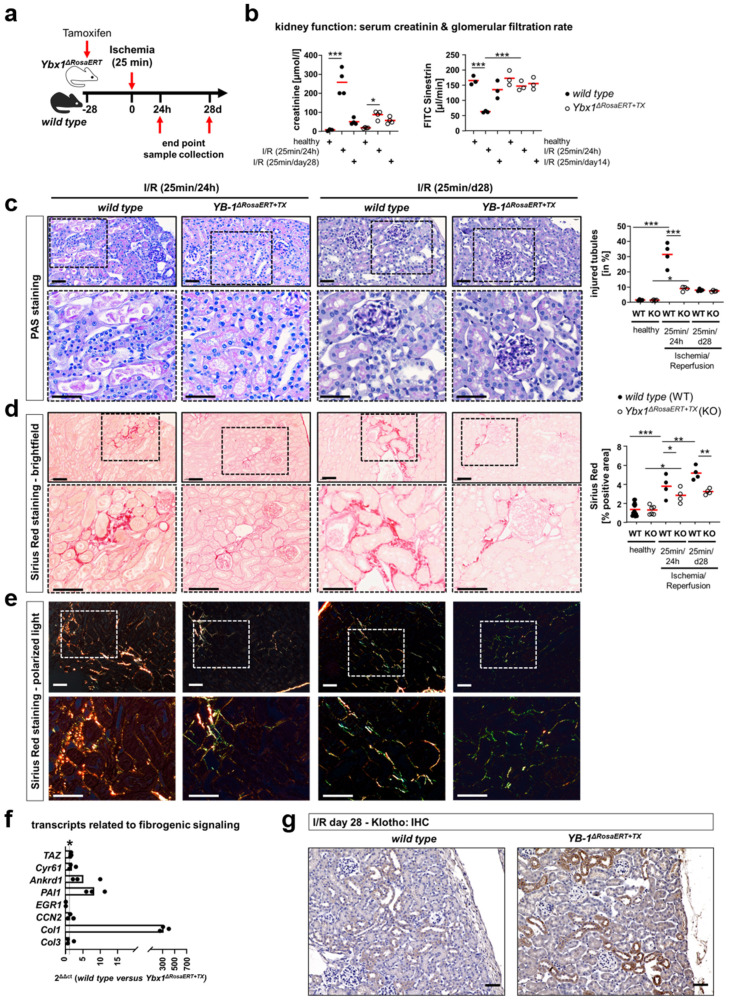
Less tubular damage and fibrogenic response in *Ybx1*-deficient animals compared to wild-type kidney tissue. (**a**) An in vivo model of experimental IRI was used with 25 min of ischemia. Kidneys were analyzed on days 1 and 28 following disease induction. (**b**) Kidney function assessed by the determination of plasma creatinine levels indicated severe AKI 1 d following IRI in wild-type animals, whereas creatinine values were unaltered in *Ybx1^ΔRosaERT+TX^* animals. Correspondingly, in wild-type animals, a decline in GFR at 24 h following disease induction is seen, and 14 days later, GFR increases. The *Ybx1^ΔRosaERT+TX^* animals showed only a slight decrease in GFR that was not increasing at day 14 following IRI. (**c**) PAS staining of kidney sections revealed increased tubular cell damage 24 h following IRI in wild-type animals compare to the kidneys of *Ybx1^ΔRosaERT+TX^* mice. On day 28 following IRI, no difference in tubular injury between wild-type and *Ybx1^ΔRosaERT+TX^* animals was seen. (**d**) Picro Sirius red staining of kidney sections at time points 1 d and 28 d following ischemia visualized fibrosis as a reddish color. (**e**) The further utilization of polarized light enabled us to determine the ratio of collagen type I (red to orange) and type III (green). Representative images are shown. Scale bar, 50 µm. (**f**) Within the kidney tissue of wild type mice, the transcripts of TAZ and the target genes thereof were increased following IRI compared to control kidneys. Amongst these, PAI1, Ankrd1, and Col1 were less abundant in the kidneys of *Ybx1^ΔRosaERT+TX^* knockout mice compared to wild-type animals 28 d following IRI. (* values > 1 represent significant changes) (**g**) Representative renal immunohistochemistry staining of Klotho in healthy and diseased kidney tissues of wild-type and *Ybx1^ΔRosaERT+TX^* animals (wild-type animals, n = 3–5; *Ybx1^ΔRosaERT+TX^* knockout mice, n = 3–5). (** *p* < 0.01 and *** *p* < 0.001).

Renal ischemia–reperfusion (I/R) injury incites acute cell damage and is a significant risk factor for chronic kidney disease (CKD). Kidneys were obtained on days 1 and 28 after I/R. During the early phase after reperfusion, the decline in kidney function in *Ybx1^ΔRosaERT+TX^* knockout mice was less pronounced than in wild-type mice (Figure 7b). Additionally, the tubular cell injury scores of the wild-type mice were significantly higher than those of the *Ybx1^ΔRosaERT+TX^* knockout mice. In a later stage of disease, the injured tubular cells within the wild-type kidney tissue showed signs of recovery (Figure 7c). Picro Sirius Red staining revealed reduced amounts of collagen in the tissue of *Ybx1^ΔRosaERT+TX^* knockout mice in the early phase after reperfusion (Figure 7d,e). Fibrogenic signaling (WNT-β-catenin, Hippo-YAP/TAZ, and TGFβ-Smad) was enhanced within wild-type kidneys compared to the kidneys of *Ybx1^ΔRosaERT+TX^* knockout mice (Figure 7f). This describes an ongoing fibrogenic response that is abrogated in the absence of *Ybx1*. Again, reduced Klotho expression correlates with enhanced fibrosis in the presence of YB-1 (i.e., wild type) (Figure 7g). We hereby defined changes in resident kidney cells in acute damage models. Subsequent analyses were performed to dissect the relative contribution of infiltrating immune cells to these changes. A previously reported animal model with the manipulation of YB-1 expression in myeloid cells was analyzed regarding the activation of the aforementioned pathways.

### 3.6. ECM Accumulation Is Orchestrated by YB-1-Expressing Tissue-Resident but Not Infiltrating Myeloid-Derived Innate Immune Cells 

**Figure 8:**
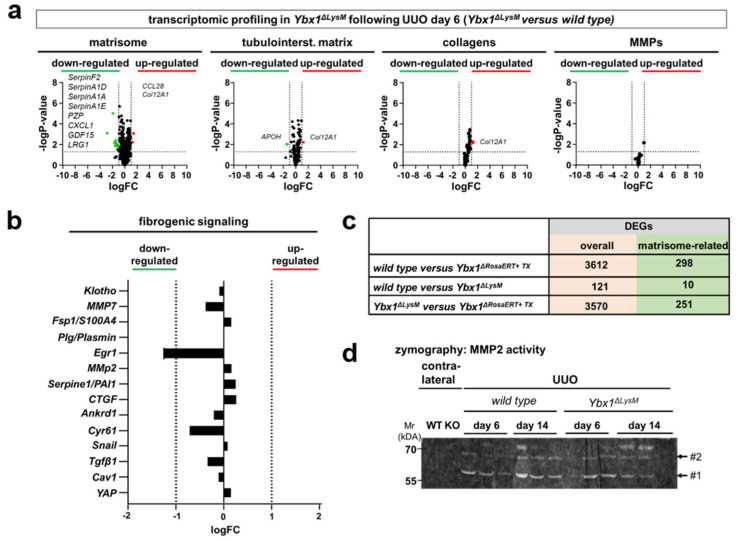
The profibrogenic response of YB-1 orchestrated by kidney-resident but not infiltrating myeloid-derived cells. Following UUO (day 6), *Ybx1^ΔLysM^* mice exhibit no obvious differences in (**a**) matrisome composition, tubulointerstitial matrix, collagen composition, MMP expression, and (**b**) fibrogenic signaling molecules between the kidneys of wild-type and *Ybx1^ΔLysM^* animals. (**c**) Quantitative comparison of DEGs between diseased kidney tissues (wild-type versus *Ybx1^ΔRosaERT+TX^* mice, wild-type versus *Ybx1^ΔLysM^* animals, and *Ybx1^ΔLysM^* versus *Ybx1^ΔRosaERT+TX^* mice). (**d**) Gelatin zymography detected MMP2 enzymatic activity in the kidney tissue lysates of wild-type and *Ybx1^ΔLysM^* animals. (#1, active MMP2; #2, latent MMP2).

To determine the contribution of infiltrating innate immune cells, we took advantage of cell-specific *Ybx1* depletion in myeloid cells (*Ybx1^ΔLysM^* strain) [37,60]. Following UUO, *Ybx1^ΔLysM^* mice exhibited enhanced tissue damage, myofibroblast activation, and fibrosis compared to wild-type animals [37]. We performed similar analyses as with whole-body *Ybx1^ΔRosaERT+TX^* knockout mice and thereby extended the previously published results. Matrisome transcriptome, collagens, MMPs, and YAP/TAZ target gene regulation revealed no obvious differences between the kidneys of healthy wild-type animals and *Ybx1^ΔLysM^* animals (Figure 8). Following UUO, the transcripts and fibrogenic signaling pathways were compared, yielding no differences. 

We compared the pro-fibrogenic phenotype in *Ybx1^ΔRosaERT+TX^* knockout and *Ybx1^ΔLysM^* mice after UUO on day 6. The differences between these two genotypes closely resemble the comparison between the wild-type and *Ybx1^ΔRosaERT+TX^* mice (Figure 8). These data indicate that cells of myeloid origin expressing YB-1 are not major contributors to profibrogenic signaling and ECM accumulation. A similar scenario was described for the Notch3 receptor. Mice lacking the receptor in myeloid cells display a strong fibrogenic response following UUO, whereas mice that lack a Notch3 receptor in tissue-resident cells displayed reduced fibrosis [43].

## 4. Discussion

Fibrosis results from excessive matrix deposition leading to the progressive loss of organ function [61,62]. The bodily response toward repair and wound healing follows a general pattern. There is an unmet medical need to understand these events and the underlying molecular decision points in order to design therapeutic interventions. Here, we identify global *Ybx1*-mediated changes in the composition and coordination of the matrisome. Using results obtained with *Ybx1*-deficient mice, we demonstrate a dominant role of YB-1 in tissue-resident cells for kidney fibrosis, both in primary tubular and glomerular injury models. By elucidating the impact of YB-1 on epigenetic modifications, particularly its influence on Klotho methylation, and identifying shared molecular pathways, this study provides novel insights into the complex mechanisms driving renal fibrosis. The findings introduce YB-1 as a pivotal player in a DNMT1–Klotho-dependent epigenetic cascade.

Matrisome-related gene composition and pro-fibrogenic signaling changes are identified in both human and mouse bulk sequencing data. Transcript regulation was linked to YB-1 binding using referenced chip-on-chip data [44], revealing a YB-1-driven matrisome in tissue-resident cells, influencing pro-fibrogenic pathways like WNT-β-catenin, Hippo-YAP/TAZ, and TGFβ-Smad (Figure 5). While the chip-on-chip data are from transformed cells, our primary cell profiling reveals potential regulatory pathways in acute-kidney-injury-induced fibrosis which may extend also to non-cancer cells (Supplementary Figure S3) [63]. Putatively, cancer cells are similarly prone to propagate a fibrogenic response with excess matrix synthesis, a phenomenon observed in solid organ cancers [63]. 

The published work underscores the importance of YB-1 in organ fibrosis [62,64,65]. Mechanistically, YB-1 fine-tunes TGFβ signaling via Smad7 and direct interactions with collagen genes and transcripts [54]. Notably, reducing YB-1 expression in *Ybx1*^±^ animals during UUO correlates with reduced tubulointerstitial fibrosis [64]. To explore YB-1’s role in the fibrogenic response to acute kidney injury, we established an inducible *Ybx1*-knockout model. We hypothesized that *Ybx1* deletion would protect against acute kidney injury and mitigate fibrosis. Consistently, this effect is observed across different injury models, suggesting a shared molecular mechanism driving fibrosis that is governed by YB-1. We identify an intriguing regulatory pathway governing fibrogenic gene expression that involves DNA methylation, typically associated with transcriptional silencing. Recent studies have highlighted the significance of DNA methylation in renal fibrosis [53,66]. Klotho is associated with reduced pro-fibrogenic responses and undergoes changes in CpG methylation in both renal patients and animal models [67,68,69]. These findings underscore the central role of DNA methylation in regulating fibrotic processes in the kidney. Cold shock protein YB-1 directs Klotho methylation. The deletion of YB-1 in mice results in reduced Klotho methylation and thereby preserves Klotho expression following injury. This observation highlights YB-1’s crucial role in suppressing Klotho expression. The DNMT1-mediated methylation of the Klotho promoter suppresses Klotho expression [42,70]. A reduced DNMT1 protein expression in *Ybx1*-deficient mice suggests a regulatory role of YB-1 in modulating DNMT1 activity. The interactome data revealed an interaction between YB-1 and DNMT1 proteins [31]. This finding suggests a YB-1-dependent recruitment of DNMT1 to Klotho, potentially explaining how YB-1 influences Klotho methylation. We further investigated YB-1’s role in Klotho methylation by analyzing the sequence of the Klotho promoter region for YB-1 binding sites, using the Y-box (inverted CCAAT) sequence as a template [29,38,48,50,54]. YB-1 is known to bind to stem-loop structures that can form due to complementary base pairing. We identified stem-loop structures using the RNAfold WebServer [56,57]. Having established the structural prerequisites for YB-1 binding to the Klotho promoter, we analyzed putative CpG motifs within the promoter region using the MethPrimer program (http://www.urogene.org/methprimer/; accessed on 15 October 2023). Several CpG sites were found in close proximity to the YB-1 binding motif (Figure 4f,g), supporting our hypothesis that YB-1 facilitates the recruitment of DNMT1 to DNA for methylation. We extended our molecular–bioinformatic approach to analyze CpG islands and YB-1 binding motifs within other genes, such as *MMP2*, *collagen type* 1, *PAI-1*, and *Snail*1. These genes are known targets of the Klotho-induced WNT/β-catenin signaling pathway and contain similar methylation motifs (Figure 4f) [53]. H3K4me2 induces methylation within the coding sequences of these genes. An inverse correlation between Klotho and PAI-1 expression in fibrotic kidneys was reported [71,72]. Here, we propose that YB-1 serves as a versatile regulator of fibrogenic gene expression by virtue of its ability to bind specific structural motifs within DNA, consequently facilitating the recruitment of methyltransferases (Figure 4h). Recently, Liu et al. showed that DNA methylation explains kidney disease heritability more than gene expression alone [73]. By performing a combined analysis of epigenetics and single-cell sequencing, they identified a similar relationship between epigenetic modifications of Klotho and fibrosis, as we report here. Other groups described a role for Klotho in progression of heart fibrosis. They proposed a model in which decreased levels of Klotho in the serum and elevated RAGE expression contribute to the activation of WNT/β-catenin signaling [74,75].

Several recent studies also utilize single-cell RNA sequencing analyses to explore the complex cellular composition of kidney tissue and its contribution to fibrosis [76,77,78,79]. These studies highlight a central role of proximal tubules in regulating kidney function [73]. Unlike bulk RNA sequencing, single-cell analysis offers insight into the cellular level of gene expression, revealing the dynamic nature of cellular diversity and heterogeneity in the fibrotic response.

**Figure 9:**
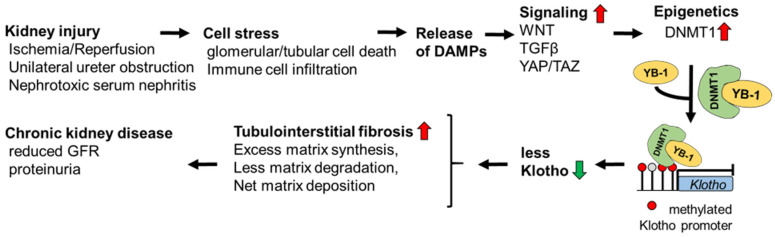
Proposed model summarizing the YB-1-dependent regulation of Klotho expression leading to chronic kidney disease. Cell stress induces the release of DAMPs, triggering WNT, TGFβ, and YAP/TAZ signaling, followed by enhanced expression of DNMT1, a protein that regulates epigenetics. The cytosolic complexes of YB-1 and DNMT1 translocate to the nucleus and bind to the Klotho promoter region. This leads to promoter hypermethylation with reduced Klotho expression. Enhanced tubulointerstitial fibrosis ensues. The deletion of YB-1 in kidney-resident cells reverses the hypermethylation of Klotho together with reduced WNT, TGFβ, and YAP/TAZ signaling. (⇧ red, enhanced activation; ⇩ green, reduced expression)Several limitations exist for our data analyses and interpretation. Our bulk sequencing data and chip-on-chip data sets should be extended by single-cell analyses. Only murine models of kidney disease have been incited. The conclusions must be transferred to the human situation and tissue analyses. It remains unclear whether the mechanisms obtained in Figure 9 apply to all cell types or are cell-specific.

In conclusion, our study defines YB-1 as a master regulator of pro-fibrogenic signaling at several mechanistic levels (Figure 9). YB-1 is a yet unreported essential part of the DNMT1–Klotho-dependent epigenetic cascade promoting renal fibrosis. Here, we propose a mechanism in which YB-1 guides DNMT1 to specific regions in the DNA of fibrosis-related genes. However, due to the multiple activities of YB-1, we cannot exclude further contributions from other mechanisms. 

## Figures and Tables

**Table 1:** Genotyping primer sequences.

Name	Sequence (5′ > 3′)	Sequence (3′ > 5′)
Rosa26Cre	GCCTGCATTACCGGTCGATGCAACGA	GTGGCAGATGGCGCGGCAACACCATT
RosaWT	CTGTGGACAGAGGAGCCATAACTGC	CCACCACTGGCTGGCTAAACTCT
LysM	CTTGGGCTGCCAGAATTTCTC	TTACAGTCGGCCAGGCTGAC
CRE8	CCCAGAAATGCCAGATTACG	
YB-1	GCCTAAGGATAGTGAAGTTTCTGG	CCTAGCACACCTTAATCTACAGCC

**Table 2:** Primer sequences for methylation-specific PCR.

Name	Sequence (5′ > 3′)	Sequence (3′ > 5′)
mKL/M	GGTATCGCGGGTATTTTTAATC	CGACATAATCCCTAAAATAATCGAC
mKL/unM	TTAATGGTATTGTGGGTATTTTTAATTG	CAACATAATCCCTAAAATAATCAAC
mIn	TAGTTTTAGGAAGGTAAAGGGAGTG	AAATCCCAAAAAAAACACAACAAA
mKL/M	GGTATCGCGGGTATTTTTAATC	CGACATAATCCCTAAAATAATCGAC

**Table 3:** Real-time PCR primer sequences.

Target (Mouse)	Sequence (5′ > 3′)	Sequence (3′ > 5′)
Col1a1	GAGAACCAGCAGAGCCA	GAACAAGGTGACAGAGGCATA
Col3a1	GAAAGGATGGAGAGTCAGGAA	GAAAGGATGGAGAGTCAGGAA
Ccn2	CCCTAGCTGCCTACCGACT	GGTAACTCGGGTGGAGATGC
Cyr61	GCTCAGTCAGAAGGCAGACC	GTTCTTGGGGACACAGAGGA
Gapdh	AGGTCGGTGTGAACGGATTTG	GGGGTCGTTGATGGCAACAA
PAI-1	GGC CAT TAC TAC GAC ATC CTG	GGT CAT GTT GCC TTT CCA GT
Egr1	CAGTCCCAGCTCATCAAAC	TCTGCTTTCTTGTCCTTCTG
DNMT1	CAGAGACTCCCGAGGACAGA	TTTACGTGTCGTTTTTCGTCTC
DNMT3a	CGTGAGTCCGGTGTGTCA	CTCCAACCACACACACAAGG
DNMT3b	TTCAGTGACCAGTCCTCAGACACGAA	TCAGAAGGCTGGAGACCTCCCTCTT

**Table 4:** Matrisome-defining genes that are linked to YB-1 binding activities.

	Immunoprecipitated Transcripts Bound by YB-1
Human Data Sets	
GSE30122	*down-regulated* : * NELL* , * SPARC* , * WISP3* , * SDC2* , * FAM20B* , * HRG* , * PLAT* , * TIMP3* , * EGF* , * FGF9* , * HBEGF* , * S100A8* , * VEGFA* *up-regulated* : * FN1* , * IGFBP3* , * COL15A1* , * C1QB* , *ST14* , * CCL19* , * HGF*
GSE116626	*down-regulated* : *SLIT3* , *COL16A1* , *CLEC4F* , * PAPPA2* , * CCL19* , * IL10* , * WNT10A* *up-regulated* : *CYR61* , * ANXA6, MMP24* , * SERPINA6* , * SERPINE1* , *ANGPTL4* , * HBEGF*
mouse data sets	
UUO	*down-regulated* : *ADIPOQ* , *IGFBP3* , * COL27A1* , * HRG* , * PZP* , * TIMP3* , * EGF* , * FGF9* , *VEGFA* *up-regulated* : * CYR61* , * FGA* , * FN1* , * IGFBP6* , * LRG1* , * MFAP2* , *MFAP5* , * MGP* , * SLIT3* , * SPARC* , * WISP1* , * COL15A1* , * COL16A1* , * COL5A1* , * COL6A2* , * ANXA5* , * ANXA6* , * C1QB* , * CLEC4D* , * LGALS3* , * MUC4* , * ADAMTS1* , * ADAMTS2* , * ITIH4* , * LOXL2* , * SERPINE1* , * ANGPTL4* , * CSF1* , * GDF15* , * HBEGF* , * IL1F6* , *S100A6* , *S100A8* , *WNT10A* , * WNT4*

(green: down-regulated in disease model; red: up-regulated in disease model).

## Data Availability

The raw data of this article will be made available by the authors on request.

## Supplementary Materials

The following supporting information can be downloaded at https://www.mdpi.com/article/10.3390/cells13050367/s1, Supplementary Figure S1. Kidney morphology and function in *Ybx1*-knockout and *wild-type* mice. Supplementary Figure S2. The UUO-dependent fibrotic signature and tubular damage differ between wild-type and *Ybx1^ΔRosaCreERT+TX^* animals. Supplementary Figure S3. Immunohistochemistry of Klotho in healthy and diseased kidney tissues of wild-type and *Ybx1^ΔRosaCreERT+TX^* animals following UUO on day 14. Supplementary Figure S4. Sheep IgG deposition within kidney tissue following the injection of NTS. Supplementary Table S1. Description of human datasets used in this study. Supplementary Table S2. Matrisome-dependent DEGs identified in samples from patients diagnosed with diabetic kidney disease and IgA nephropathy. Supplementary Table S3. Differential expression genes defining the matrisome in *Ybx1^ΔRosaERT+TX^* knockout animals compared to wild-type animals following UUO. Uncropped Western blot pictures.
