# Supplementary material for: Comparative Analysis of Acute Kidney Injury Models and Related Fibrogenic Responses: Convergence on Methylation Patterns Regulated by Cold Shock Protein
Source: Cells. 2024 Feb 20;13(5):367. doi: 10.3390/cells13050367 (PMC10930537; doi:10.3390/cells13050367)
Supplement: Supplementary file 1 [file cells-13-00367-s001.zip › cells-2825059-supplementary.pdf]

Supplemental Material

Supplementary Figure S1. Kidney morphology and function of *Ybx1*-knockout and *wild type* mice.
Supplementary Figure S2. The UUO-dependent fibrotic signature and tubular damage differ between wild type and $Ybx1^{\Delta RosaCreERT+TX}$ animals.
Supplementary Figure S3. Immunhistochemistry of Klotho in healthy and diseased kidney tissue of wild type and $Ybx1^{\Delta RosaCreERT+TX}$ following UUO on day 14.
Supplementary Figure S4. Sheep IgG deposition within kidney tissue following injection of NTS.

Supplementary Table S1. Description of human datasets used in the study.
Supplemntary Table S2. Matrisome-dependent DEGs identified in samples of patients diagnosed with diabetic kidney disease and IgA nephropathy.
Supplemntary Table S3. Differential expression genes defining the matrisome in $Ybx1^{\Delta RosaERT+TX}$ knock out compared to wild type animals following UUO.

Uncroped Western Blot pictures.

Supplementary Figure S1

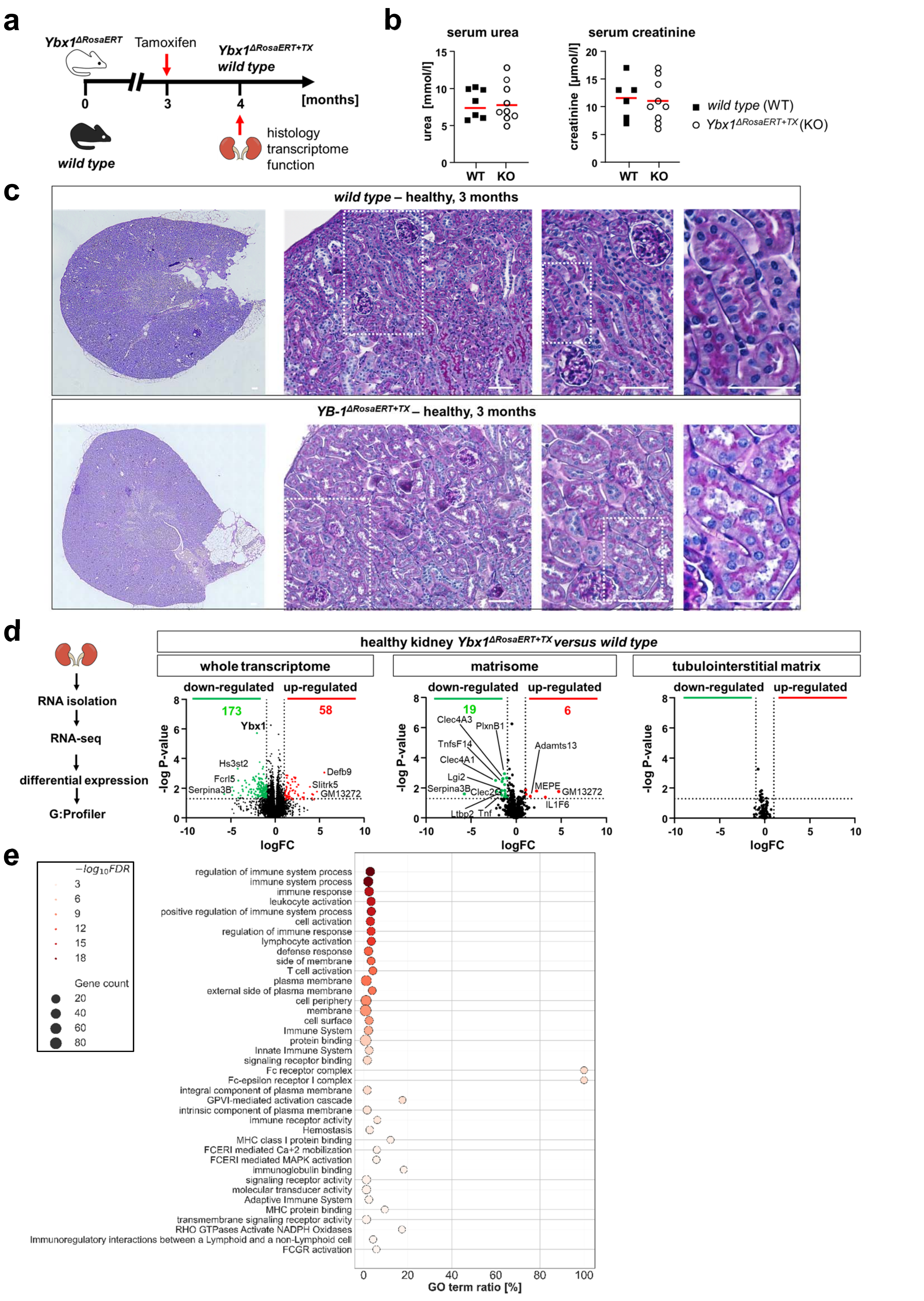

Supplementary Figure S1. Kidney morphology and function of *Ybx1*-knockout and *wild type* mice. (A Tamoxifen was applied in 8 week old mice. 28 days later kidneys of wild type (n=6 and *Ybx1*-knockout (n=8 mice were analyzed. No differences between wild type and *Ybx1*-knockout animals were seen for **(B** kidney function (blood urea nitrogen (BUN, serum creatinine and **(C** kidney morphology (Periodic Acid-Schiff (PAS staining, scale bars equal 5 μm. **(D** Volcano plot of differentially expressed genes of *Ybx1*-knockout versus *wild type*. The differences according the whole RNAseq data, to the matrisome relevant genes, and to the tubulointerstitial matrix are depicted. The most significantly upregulated genes in *Ybx1*-knockout mice are shown in red, whereas green represents the significantly downregulated genes. Black dots are genes that are differentially expressed below the cutoff values of log-fold change and -log$_1$ of the P-value. Significantly regulated transcripts were those with a logFC ≤ ±1 and -log$_1$ P-value >1.3 (dotted lines . **(E** Gene enrichment analysis of the differentially expressed genes between healthy *Ybx1*-knockout and wild type kidney tissue. The size of the dots reflects the gene counts for each process and the dot color the P-values, where the most significant result is purple.

Supplementary Figure S2

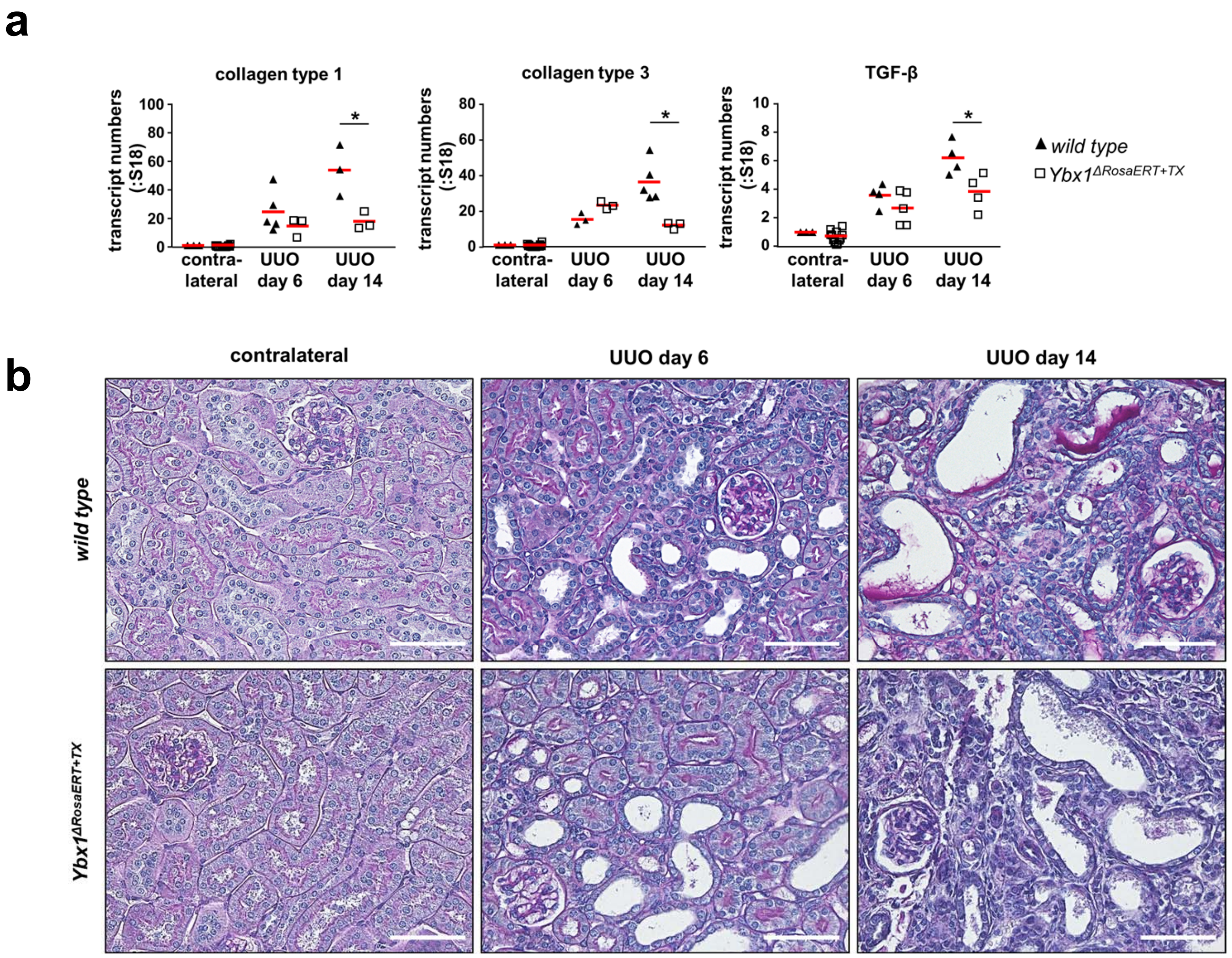

Supplementary Figure S2. The UUO-dependent fibrotic signature and tubular damage differ between wild type and *Ybx1*$^{\Delta RosaCreERT+TX}$ animals. (a)TaqMan analysis of *Col1a1*, *Col1a3*, and *TGFβ* transcript numbers in healthy contralateral and obstructed kidneys. Both wild type and *Ybx1*$^{\Delta RosaCreERT+TX}$ animals revealed an upregulation of the fibrogenic genes; however to a different degree. **(b** Periodic acid-Schiff (PAS staining of cortical kidney tissue was performed and representative images were taken. The PAS stainings of obstructed kidneys at days 6 and 14 show efficient congestion of the kidneys in both genotypes. Scale bar, 5 µm. (wild type n=3-5; *Ybx1*$^{\Delta RosaCreERT+TX}$ n=3-5)

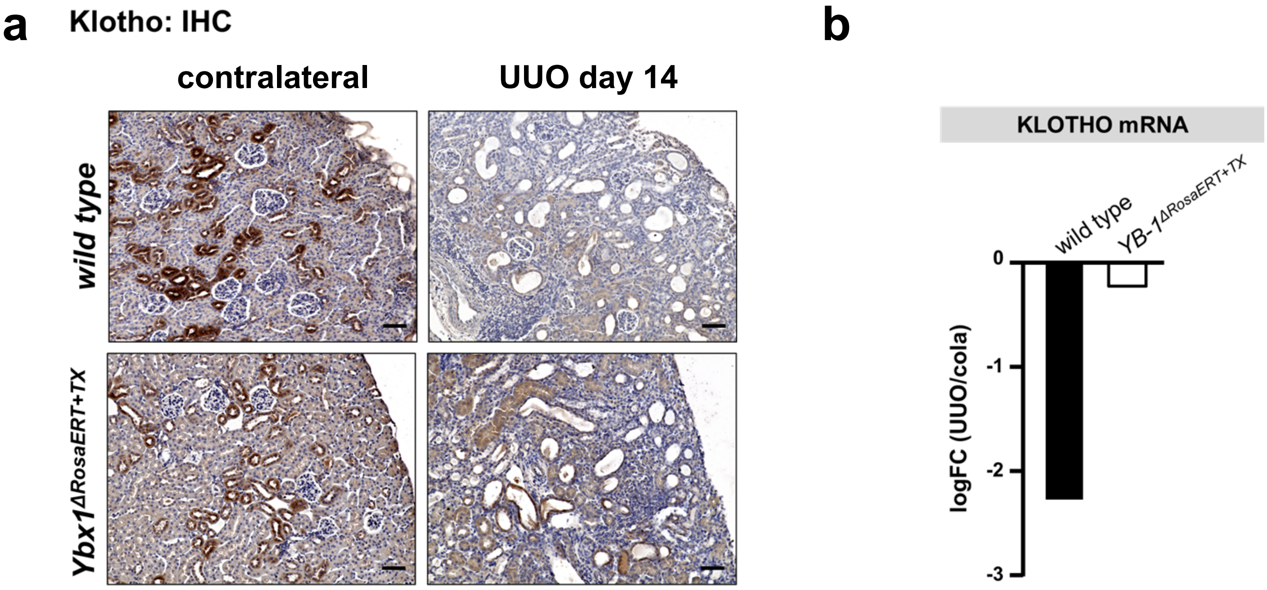

Supplementary Figure S3. KLOTHO is regulated on translational and transcriptional level in a YB-1-dependent manner. (a Immunhistochemistry of Klotho in healthy and diseased kidney tissue of wild type and *Ybx1*$^{\Delta RosaCreERT+TX}$ following UUO on day 14. **(b)** Reduced KLOTHO transcripts in wild type kidney tissue following UUO.

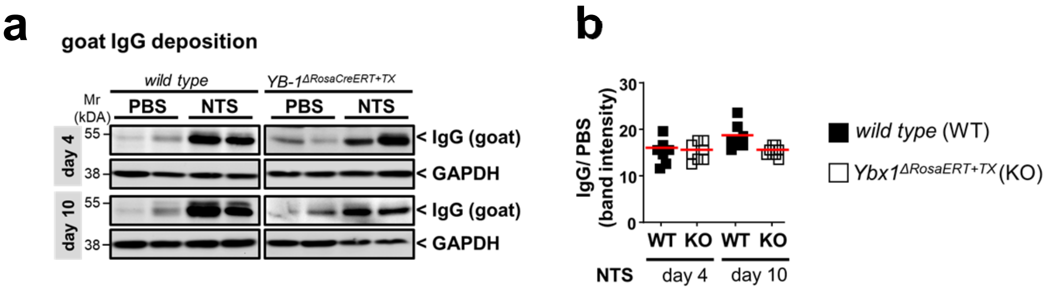

Supplementary Figure S4. Sheep IgG deposition within kidney tissue following injection of NTS. (a) Kidney tissue of wild type and *Ybx1*$^{\Delta RosaCreERT+TX}$ animals was analyzed for sheep IgG deposition following NTS day 4 and day 10. **(b)** Quantification of the IgG band intensitiy was performed according to PBS injected animals. (wild type n=6; *Ybx1*$^{\Delta RosaCreERT+TX}$ n=6)

Supplementary Table S1. Description of human datasets used in the study.

GEO series	plattform	model	Ref.
**GSE30122**	GPL571 Affymetrix Human Genome U133A 2.0	diabetic kidney disease	[35]
**GSE116626**	GPL14951 Illumina HumanHT-12 WG-DASL V4.0 R2	IgA nephropathy	[36]

35. Woroniecka KI, Park AS, Mohtat D, *et al.* Transcriptome analysis of human diabetic kidney disease. *Diabetes* 2011; **60:** 2354-2369.

36. Cox SN, Chiurlia S, Divella C, *et al.* Formalin-fixed paraffin-embedded renal biopsy tissues: an underexploited biospecimen resource for gene expression profiling in IgA nephropathy. *Scientific reports* 2020; **10:** 15164.

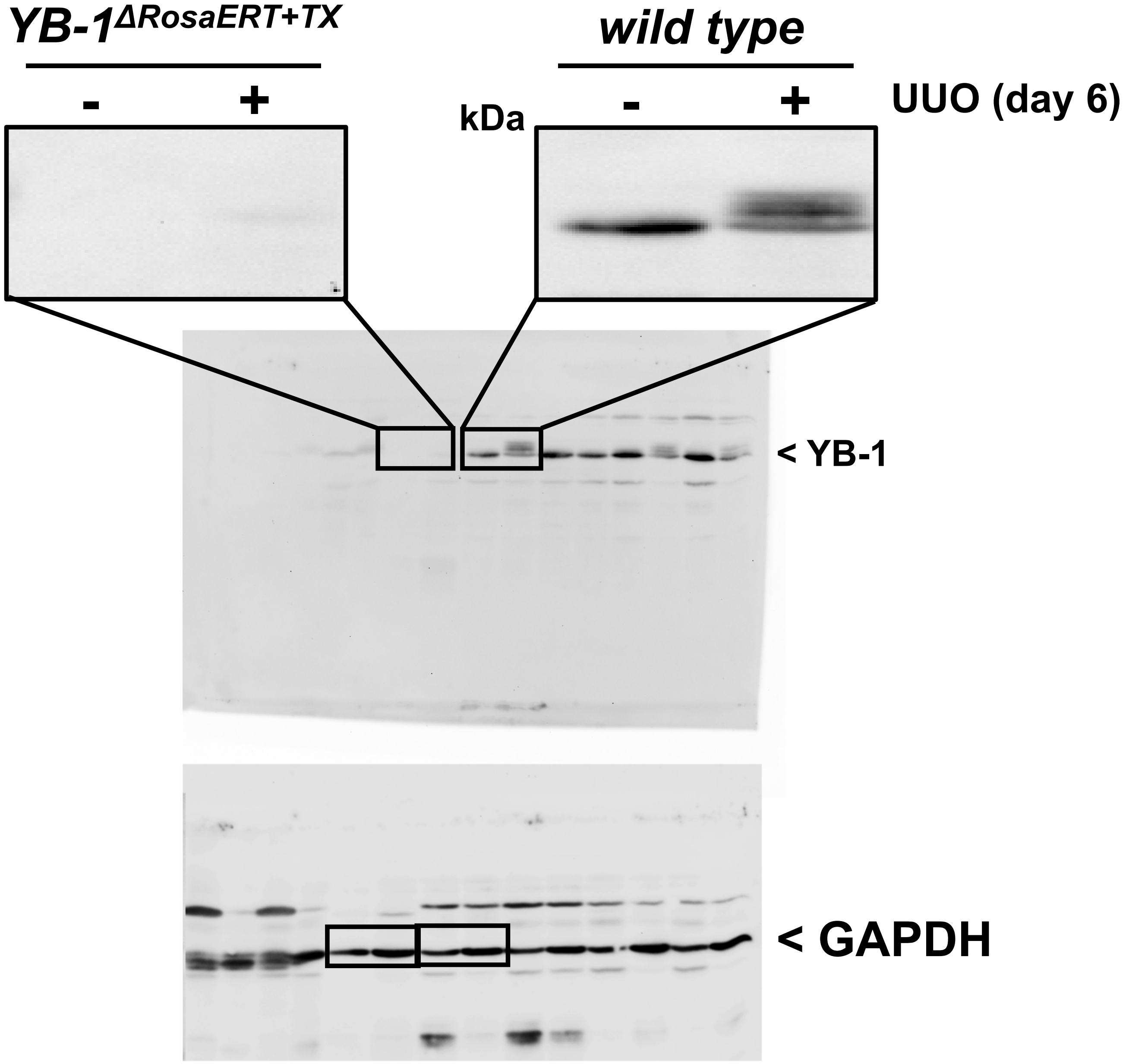

YB-1ΔRosaERT+TX
wild type
- +
kDa
- +
UUO (day 6)
< YB-1
< GAPDH

zymography

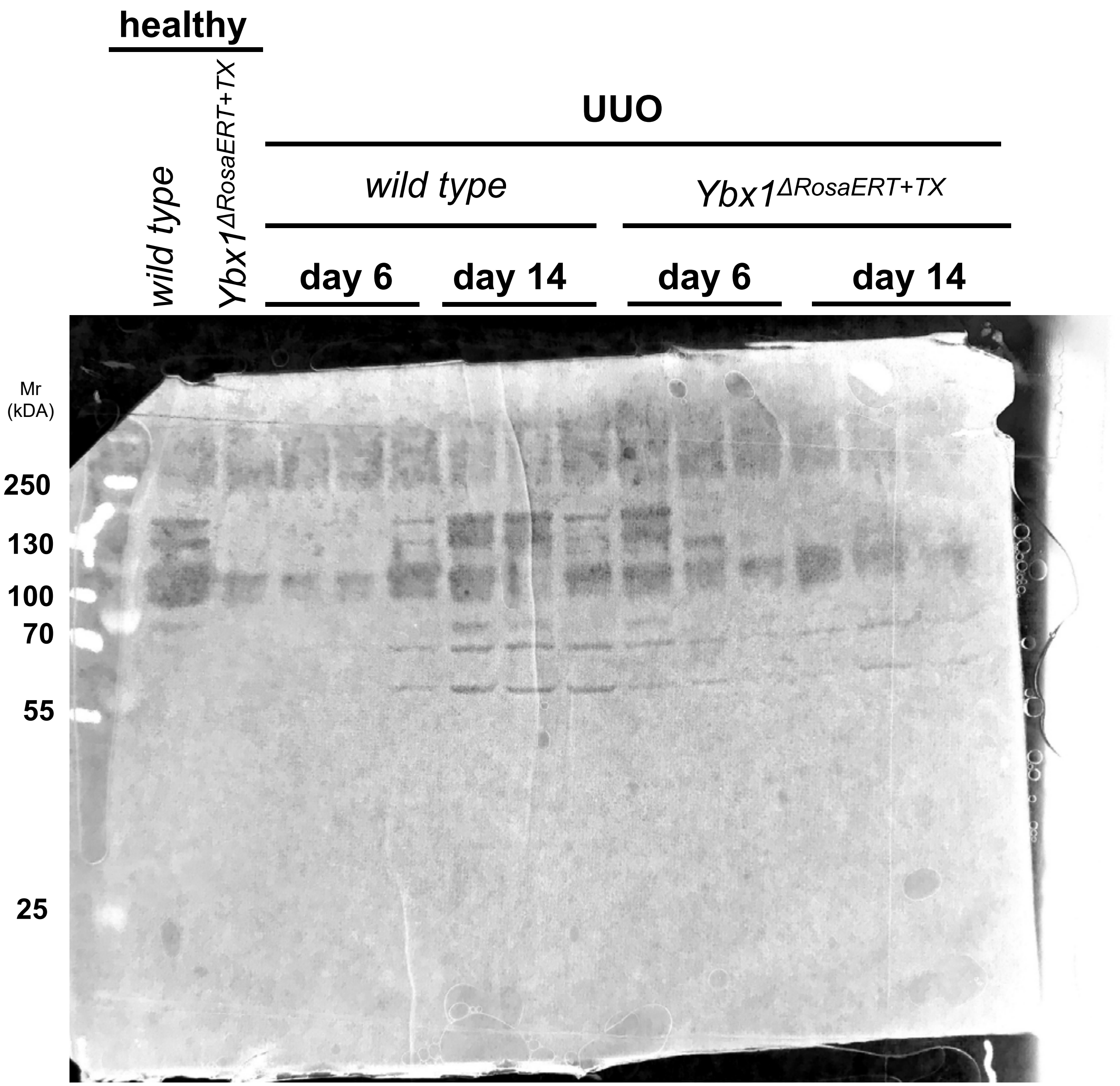
healthy
wild type
Ybx1ΔRosaERT+TX
UUO
wild type
Ybx1ΔRosaERT+TX
day 6
day 14
day 6
day 14
Mr
(kDA)
250
130
100
70
55
25

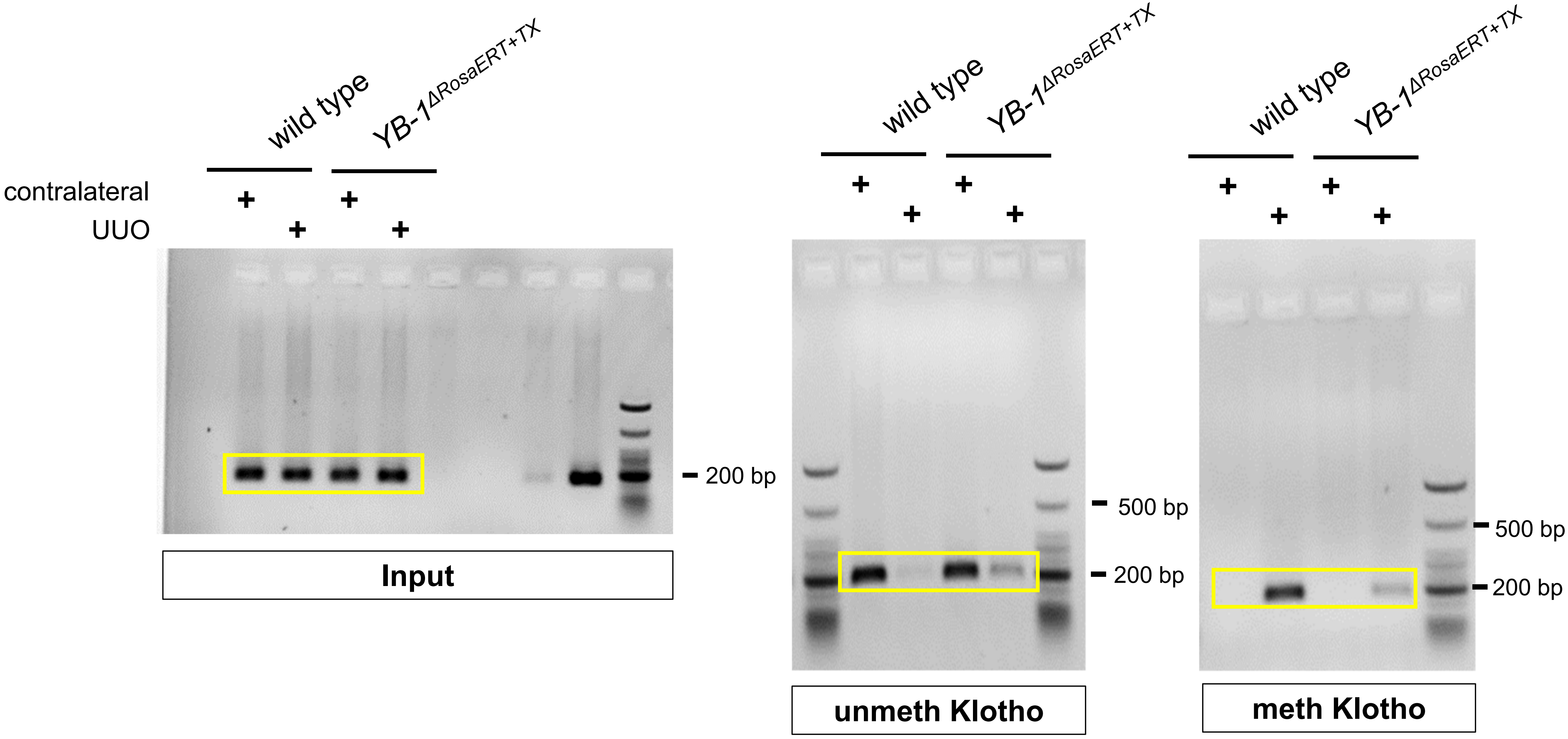

wild type
YB-1 ΔRosaERT+TX
contralateral
UUO
+
200 bp
Input
500 bp
200 bp
unmeth Klotho
500 bp
200 bp
meth Klotho

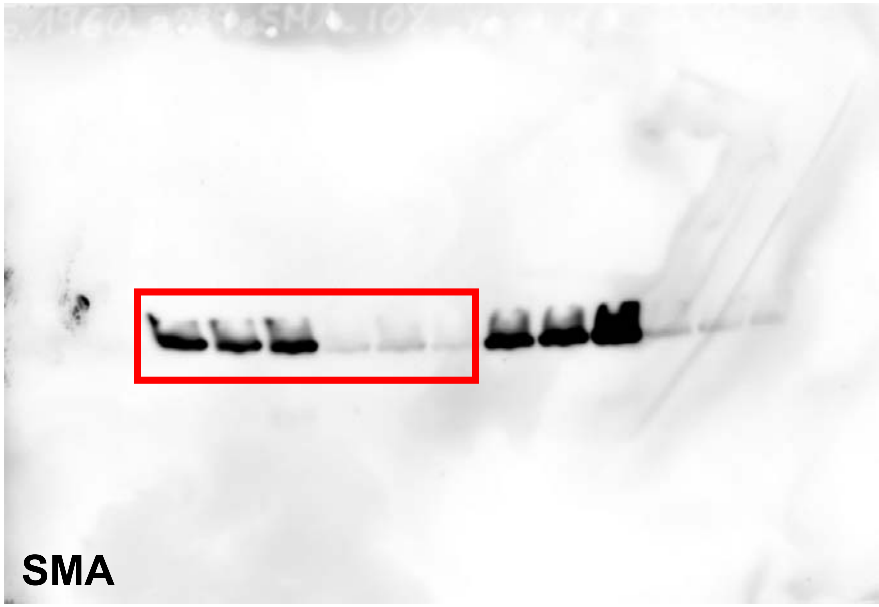
SMA

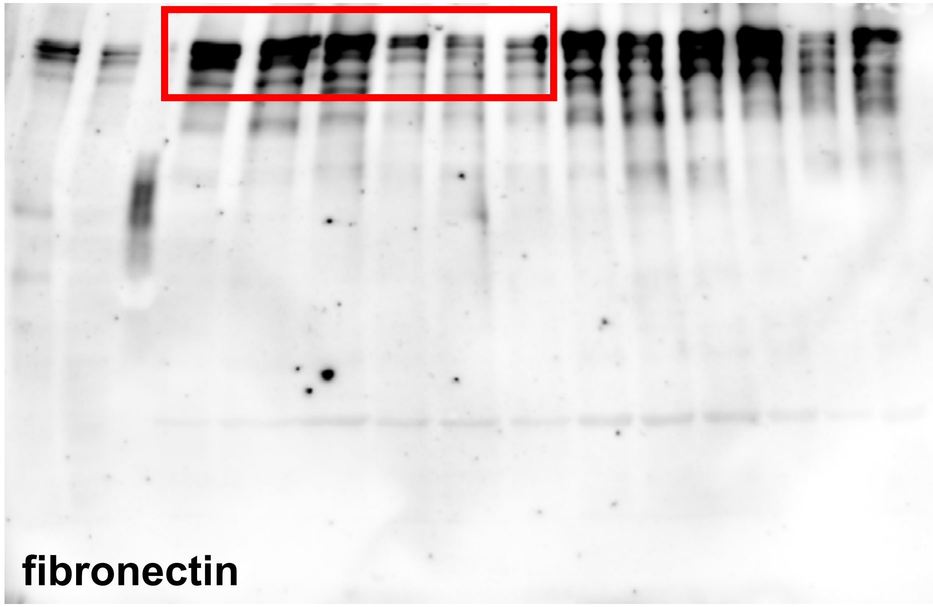
fibronectin

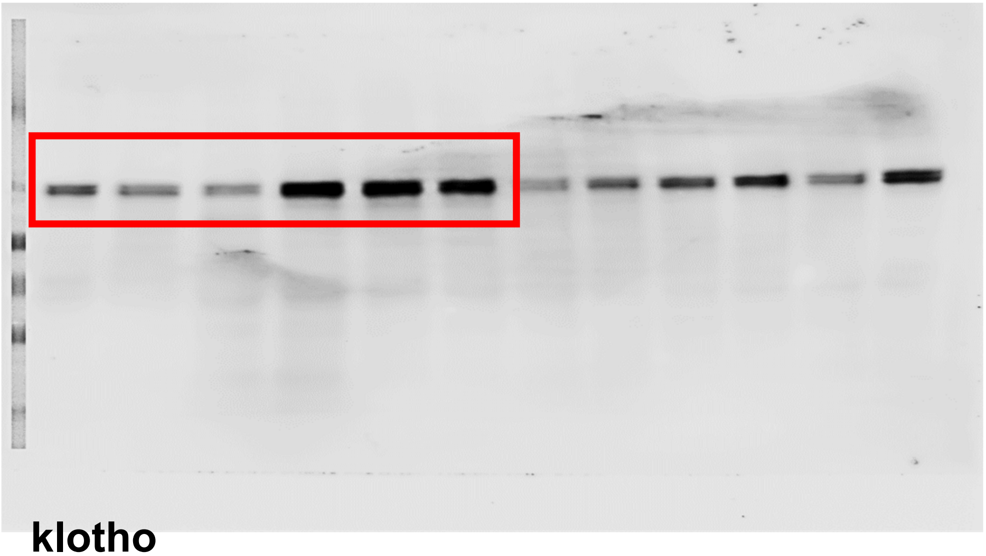
klotho

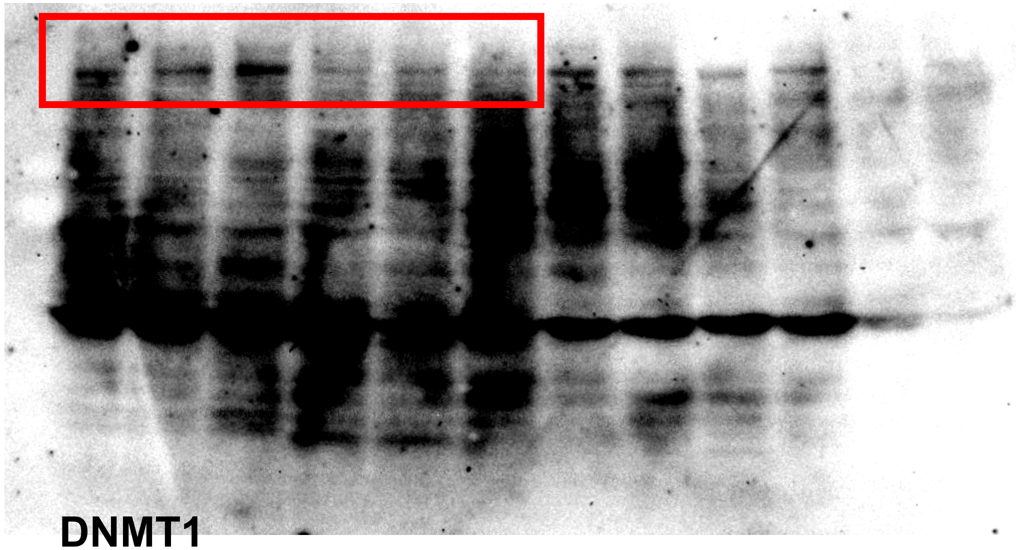
DNMT1

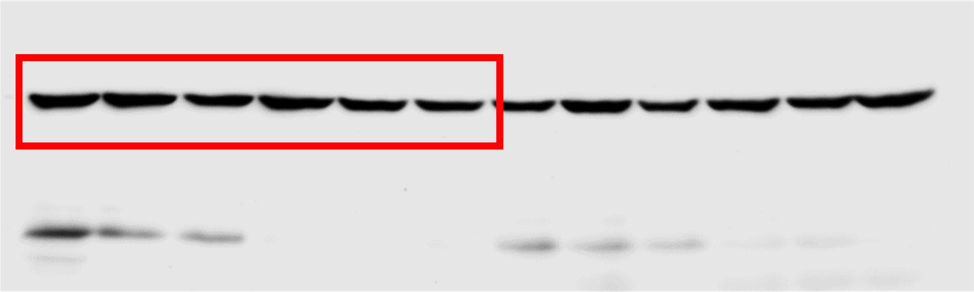

vinculin

zymography

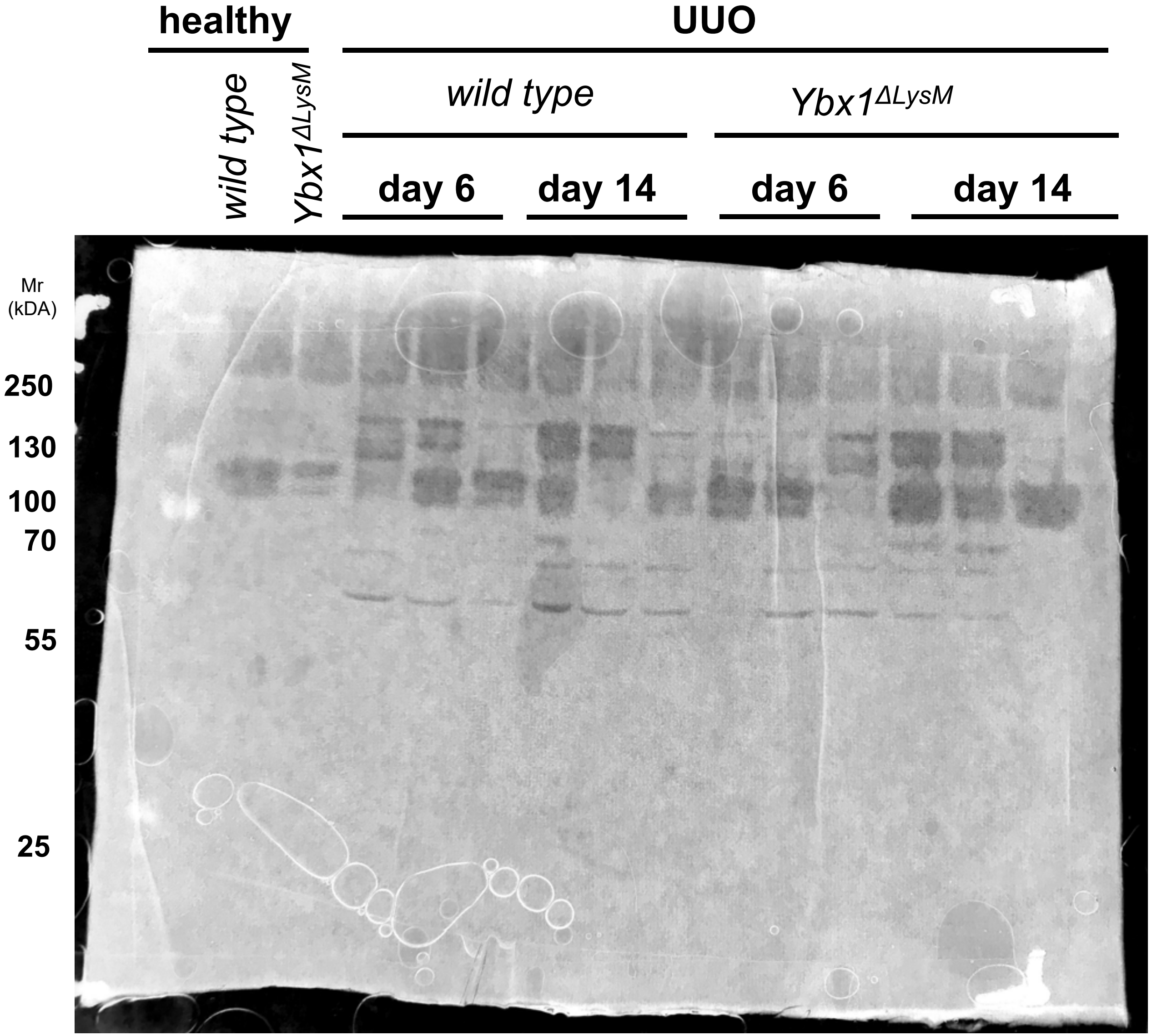
healthy
UUO
wild type
Ybx1ΔLysM
wild type
Ybx1ΔLysM
day 6
day 14
day 6
day 14
Mr
(kDA)
250
130
100
70
55
25
